# Supplementary material for: Going beyond gadgets: the importance of scalability for analogue quantum simulators
Source: Nat Commun. 2024 Aug 2;15:6527. doi: 10.1038/s41467-024-50744-9 (PMC11297168; doi:10.1038/s41467-024-50744-9)
Supplement: Supplementary file 1 — Supplementary Information [file 41467_2024_50744_MOESM1_ESM.pdf]

Going beyond gadgets: The importance of scalability for analogue quantum simulators

Dylan Harley, Frederik Ravn Klausen, Ishaun Datta, Andreas Bluhm, Daniel Stilck França, Albert H. Werner, and Matthias Christandl

Supplementary Note 1 — Local encodings

In this section we prove the simple results concerning local encodings.

Proposition 1 (Restatement of Proposition 7 in the Main Text). *Let $\mathcal{E}_{\text{state}} : D(\mathcal{H}) \to D(\mathcal{H}')$ be a local state encoding, and let $\mathcal{N}' : D(\mathcal{H}') \to D(\mathcal{H}')$ be a channel whose Kraus operators $\{X'_k\}$ each act on $O(1)$ sites in $\mathcal{H}'$. Then there exists a channel $\mathcal{N} : D(\mathcal{H} \otimes F) \to D(\mathcal{H} \otimes F)$ whose Kraus operators $\{X_k\}$ each act on $O(1)$ sites in $\mathcal{H} \otimes F$, and such that for all $\rho \in \mathcal{H}$,*

$$\mathcal{N}' \circ \mathcal{E}_{\text{state}}(\rho) = \text{tr}_E[U\mathcal{N}(\rho \otimes |0\rangle\langle 0|_F)U^\dagger] \ . \tag{1}$$

Proof of Proposition 1. We prove this for a single Kraus operator X'_k acting on $O(1)$ sites in $\mathcal{H}'$, and the result follows by linearity. Note that

$$\begin{aligned} X'_k \mathcal{E}_{\text{state}}(\rho)(X'_k)^\dagger &= X'_k \,\text{tr}_E[U(\rho \otimes |0\rangle\langle 0|_F)U^\dagger](X'_k)^\dagger \\ &= \text{tr}_E[UX_k(\rho \otimes |0\rangle\langle 0|_F)X_k^\dagger U^\dagger] \ , \end{aligned} \tag{2}$$

where $X_k = U^\dagger(X'_k \otimes \text{I}_E)U$, which acts on $O(1)$ sites in $\mathcal{H} \otimes F$ by the causality assumptions on U.

∎

Proposition 2 (Restatement of Proposition 9 in the Main Text). *Let $\mathcal{E}_{\text{obs}}$ be a local observable encoding, and let O be a local operator on $\mathcal{H}$. Then $\mathcal{E}_{\text{obs}}(O)$ can be measured using a local POVM on $\mathcal{H}'$.*

Proof of Proposition 2. To see this, we use the definition of local state encodings and write

$$\mathcal{E}^*_{obs}(\rho') = \text{tr}_G[W(\rho' \otimes |0\rangle\langle 0|_E)W^\dagger] \ , \tag{3}$$

where $W \in \text{U}(\mathcal{H}' \otimes E, \mathcal{H} \otimes G)$ is a constant-depth quantum circuit. Then the measurement expectation value is

$$\begin{aligned} \text{tr}[\mathcal{E}_{\text{obs}}(O)\rho'] &= \text{tr}[O\mathcal{E}^*_{\text{obs}}(\rho')] \\ &= \text{tr}[(O \otimes \text{I}_G)W(\rho' \otimes |0\rangle\langle 0|_E)W^\dagger] \\ &= \text{tr}[(\text{I}_{\mathcal{H}'} \otimes \langle 0|_E)W^\dagger(O \otimes \text{I}_G)W(\text{I}_{\mathcal{H}'} \otimes |0\rangle_E)\rho'] \ . \end{aligned} \tag{4}$$

Assuming O is local, then $W^\dagger(O \otimes \text{I}_G)W$ acts only on a constant-sized subsystem of $\mathcal{H}'$. In particular, we can write $\mathcal{H}' = A \otimes A^c$ where A consists of $O(1)$ sites, and then

$$W^\dagger(O \otimes \text{I}_G)W = O' \otimes \text{I}_{A^c} \ , \tag{5}$$

for some O' acting on $A \otimes E$. Then

$$\mathrm{tr}[\mathcal{E}_{\mathrm{obs}}(O)\rho'] = \mathrm{tr}[(\mathrm{I}_A \otimes \langle 0|_E)O'(\mathrm{I}_A \otimes |0\rangle_E)\rho'_A] \ , \tag{6}$$

which can be estimated via a POVM on A. ∎

Supplementary Note 2 — Qutrit-to-qubit energy scaling

Here we prove the following proposition about qutrit-to-qubit encodings.

Proposition 3 (Restatement of Proposition 10 in the Main Text)**.** *Let $\mathcal{H} = (\mathbb{C}^3)^{\otimes n}$ be the space of n qutrits acted on by the Hamiltonian*

$$H_n = \sum_{j=1}^{n} (P_0^{(j)} + P_\uparrow^{(j)}) \ . \tag{7}$$

Suppose $H'_n = \sum_{j=1}^{K} h'_j$ is a k-local Hamiltonian on $\mathcal{H}' = (\mathbb{C}^2)^{\otimes m}$, where $m = O(n^{1+\alpha})$, for $\alpha \geq 0$ and $k = O(1)$. Assume the interaction hypergraph of H'_n has degree bounded by $d = O(1)$.

If H'_n is a (Δ, η, ϵ)-simulation for H_n in the sense of Cubitt et al.[1], for $\eta \in [0, 1)$ and $\epsilon \geq 0$, then

$$\max_j \|h'_j\| = \Omega(n^{1-\alpha}(1 - \eta^2)) \ . \tag{8}$$

The idea is simple: by encoding a qutrit into a set of qubits, we must end up with an "unused" state in the qubit system, since the encoding cannot be surjective by dimension counting. Since the (Δ, η, ϵ) simulation requires all simulated states to lie in the low-energy subspace of the simulator, this implies that the unused qubit states must lie in the high-energy (above Δ) subspace.

In the proof below, we start with the encoded ground state ρ_0, and construct a state ρ_1 which differs only from ρ_0 only in one set of qubits in which it is in such an "unused" state. The similarity of the states and their differences in energies lead to the requirement for strong interactions.

In this proof, and subsequent sections, we make frequent use of the following standard result from matrix analysis[2].

Lemma 4 (Weyl's Perturbation Theorem)**.** *Let $A, B \in \mathrm{Herm}(\mathcal{H})$ be Hermitian matrices, with spectra $\lambda_0 \leq \lambda_1 \leq \ldots$ and $\mu_0 \leq \mu_1 \leq \ldots$ respectively. Then*

$$\max_j |\lambda_j - \mu_j| \leq \|A - B\| \ . \tag{9}$$

Proof of Proposition 3. Write $\mathcal{H} = \otimes_{i=1}^{n} \mathcal{H}_i$, where $\mathcal{H}_i = \mathbb{C}^3$ is a single qutrit site. By the definition of local simulation given by Cubitt et al.[1], we have two encodings $\mathcal{E}_{\mathrm{obs}}$ and $\tilde{\mathcal{E}}_{\mathrm{obs}}$ of the form (using that H is real to set $Q = 0$ without loss of generality)

$$\mathcal{E}_{\mathrm{obs}}(M) = V(M \otimes P)V^\dagger \ , \quad \tilde{\mathcal{E}}_{\mathrm{obs}}(M) = \tilde{V}(M \otimes P)\tilde{V}^\dagger \ , \tag{10}$$

where P is a projector on the ancillary space $\mathcal{A}$, and $V, \tilde{V}$ are both isometries $\mathcal{H} \otimes \mathcal{A} \to \mathcal{H}'$. These encodings satisfy the properties:

- $\mathcal{E}_{\text{obs}}$ is a local encoding, in the sense that $\mathcal{A} = \otimes_{i=1}^n \mathcal{A}_i$ and $V = \otimes_{i=1}^n V_i$ where $V_i : \mathcal{H}_i \otimes \mathcal{A}_i \to \mathcal{H}'_i$. Here we write $\mathcal{H}'_i \cong (\mathbb{C}^2)^{\otimes m_i}$ for the set of m_i qubits into which qutrit i is encoded. Note $\sum_i m_i = m$.
- $\tilde{\mathcal{E}}_{\text{obs}}$ satisfies
$$\tilde{\mathcal{E}}_{\text{obs}}(\mathrm{I}) = \tilde{V}(\mathrm{I} \otimes P)\tilde{V}^\dagger = P_{\leq\Delta(H'_n)} \ , \tag{11}$$
where $P_{\leq\Delta(H'_n)}$ is the low-energy (below Δ) projector for H'_n, and
$$\|P_{\leq\Delta(H'_n)} H'_n P_{\leq\Delta(H'_n)} - \tilde{\mathcal{E}}_{\text{obs}}(H_n)\| \leq \epsilon \ . \tag{12}$$
- $\mathcal{E}_{\text{obs}}$ and $\tilde{\mathcal{E}}_{\text{obs}}$ are close, in the sense that
$$\|V - \tilde{V}\| \leq \eta \ . \tag{13}$$

Now we define a state $\tau \in \text{span}(P)$ and define a state encoding (in the sense of Cubitt et al.[1])
$$\tilde{\mathcal{E}}_{\text{state}}(\rho) = \tilde{V}(\rho \otimes \tau)\tilde{V}^\dagger \ . \tag{14}$$
Let $\rho_0 = \tilde{\mathcal{E}}_{\text{state}}(|{\downarrow}\rangle\langle{\downarrow}|^{\otimes n})$ be the encoded ground state of H_n, which by definition satisfies
$$P_{\leq\Delta(H'_n)}\rho_0 = \rho_0 \ , \quad \tilde{\mathcal{E}}_{\text{obs}}(H_n)\rho_0 = 0 \ . \tag{15}$$
Hence we can bound the energy of ρ_0 under H'_n by
$$\begin{aligned} \mathrm{tr}[H'_n\rho_0] &= \mathrm{tr}[P_{\leq\Delta(H'_n)} H'_n P_{\leq\Delta(H'_n)}\rho_0] \\ &= \mathrm{tr}[(P_{\leq\Delta(H'_n)} H'_n P_{\leq\Delta(H'_n)} - \tilde{\mathcal{E}}_{\text{obs}}(H_n))\rho_0] \\ &\leq \|P_{\leq\Delta(H'_n)} H'_n P_{\leq\Delta(H'_n)} - \tilde{\mathcal{E}}_{\text{obs}}(H_n)\| \\ &\leq \epsilon \ . \end{aligned} \tag{16}$$
Now without loss of generality we assume that $m_1 = \min_i m_i$. Notice that $V_1 : \mathcal{H}_1 \otimes \mathcal{A}_1 \to \mathcal{H}'_1$ cannot be surjective, since
$$\dim(\mathcal{H}_1 \otimes \mathcal{A}_1) = 3\dim\mathcal{A}_1 \neq 2^{m_1} \ . \tag{17}$$
We can therefore choose some pure state $\psi = |\psi\rangle\langle\psi|$ in $\mathcal{H}'_1$ which is orthogonal to the image of V_1, and define
$$\rho_1 = \psi \otimes \mathrm{tr}_1[\rho_0] \in \mathrm{Lin}(\mathcal{H}') \ , \tag{18}$$
Where tr_1 denotes the partial trace over the $\mathcal{H}'_1$ system. This satisfies $V^\dagger\rho_1 = \rho_1 V = 0$, so we have
$$\begin{aligned} \mathrm{tr}[P_{\leq\Delta(H'_n)}\rho_1] &= \mathrm{tr}[(\mathrm{I} \otimes P)\tilde{V}^\dagger\rho_1\tilde{V}] \\ &\leq \mathrm{tr}[\tilde{V}^\dagger\rho_1\tilde{V}] \\ &= \mathrm{tr}[(\tilde{V} - V)^\dagger\rho_1(\tilde{V} - V)] \\ &\leq \|(\tilde{V} - V)(\tilde{V} - V)^\dagger\| \\ &\leq \eta^2 \ , \end{aligned} \tag{19}$$
from which we deduce that
$$\mathrm{tr}[H'_n\rho_1] \geq \Delta\,\mathrm{tr}[(\mathrm{I} - P_{\leq\Delta(H'_n)})\rho_1] - \epsilon\,\mathrm{tr}[P_{\leq\Delta(H'_n)}\rho_1] \geq \Delta(1 - \eta^2) - \epsilon\eta^2 \ , \tag{20}$$

using that the smallest eigenvalue of H'_n is at least $-\epsilon$, by (12) and Lemma 4. Therefore, using (16),

$$\mathrm{tr}[H'_n(\rho_1 - \rho_0)] \geq \Delta(1-\eta^2) - \epsilon(1+\eta^2)\ . \tag{21}$$

On the other hand, by expanding H'_n we can write

$$\mathrm{tr}[H'_n(\rho_1 - \rho_0)] = \sum_{j=1}^{K} \mathrm{tr}[h'_j(\rho_1 - \rho_0)]\ . \tag{22}$$

Notice that if h'_j acts trivially on $\mathcal{H}'_1$, that is $h'_j = \mathrm{I}_1 \otimes \tilde{h}_j$, then

$$\begin{aligned}\mathrm{tr}[h'_j(\rho_1 - \rho_0)] &= \mathrm{tr}[(\mathrm{I}_1 \otimes \tilde{h}_j)(\psi \otimes \mathrm{tr}_1\,\rho_0 - \rho_0)] \\ &= \mathrm{tr}_1[\psi]\,\mathrm{tr}_{2,3,\ldots}[\tilde{h}_j\,\mathrm{tr}_1[\rho_0]] - \mathrm{tr}[(\mathrm{I} \otimes \tilde{h}_j)\rho_0] \\ &= 0\ .\end{aligned} \tag{23}$$

Hence the only non-zero contributions to (22) come from j in the set

$$I_1 = \{1 \leq j \leq K \mid h'_j \text{ acts non-trivially on } \mathcal{H}'_1\}\ . \tag{24}$$

So (22) can be bounded by

$$\mathrm{tr}[H'_n(\rho_1 - \rho_0)] = \sum_{j \in I_1} \mathrm{tr}[h'_j(\rho_1 - \rho_0)] \leq 2|I_1| \max_{j \in I_1} \|h'_j\|\ , \tag{25}$$

using the Hölder inequality for Schatten p-norms.

Now notice that, since the largest eigenvalue of H_n is n, and the encoding $\tilde{\mathcal{E}}_{\mathrm{obs}}$ preserves spectra, we have

$$\|\tilde{\mathcal{E}}_{\mathrm{obs}}(H_n)\| = \|H_n\| = n\ , \tag{26}$$

so by (12) and Lemma 4

$$\|P_{\leq \Delta(H'_n)} H'_n P_{\leq \Delta(H'_n)}\| \geq n - \epsilon\ . \tag{27}$$

Hence, by the definition of $P_{\leq \Delta(H'_n)}$, we must have $\Delta > n - \epsilon$. Combining this fact with (21) and (25), we deduce that

$$\max_{j \in I_1} \|h'_j\| > \frac{1}{2|I_1|}\big((n-\epsilon)(1-\eta^2) - \epsilon(1+\eta^2)\big)\ . \tag{28}$$

Finally, note that $m_1 \leq m/n = O(n^\alpha)$ and $|I_1| \leq d m_1$, so for large n we have the desired scaling

$$\max_{j \in I_1} \|h'_j\| \geq \Omega\big(n^{1-\alpha}(1-\eta^2)\big)\ . \tag{29}$$

■

Lemma 5 (Restatement of Lemma 11 in the Main Text)**.** *Let $\{x_i\}_{i=1}^n$ be the points in a hypercube of side length $L \sim n^{1/D}$ in the square lattice $x_i \in \mathbb{Z}^D$. Let $\mathcal{E} : x_i \mapsto X_i \subseteq \mathbb{Z}^D$ be a map which encodes each point x_i into a connected set of points in $\mathbb{Z}^D$ such that $|X_i| = \Omega(n^\alpha)$ and $X_i \cap X_j = \emptyset$. Let $d(x,y) : \mathbb{Z}^D \times \mathbb{Z}^D \to \mathbb{Z}$ be the taxicab metric on $\mathbb{Z}^D$.*

For a radius $R = O(L)$, and any $y \in \mathbb{Z}^D$, the number of encoded points intersecting with the ball of

radius R centred at y is bounded by

$$|B_R(y)| := |\{X_i : \exists x \in X_i \text{ with } d(x,y) \leq R\}| = O\big(n^{1-\min\{\alpha,1/D\}}\big) \ . \tag{30}$$

Proof of Lemma 5. A ball of radius R contains $O(R^D)$ points in $\mathbb{Z}^D$, so at most $O(R^D/n^\alpha) = O(n^{1-\alpha})$ of the X_i can be fully contained within.

If an X_i is partially contained within the ball, then it must intersect with its boundary. There are only $O(R^{D-1})$ points on the boundary, so this can be the case for $O(R^{D-1}) = O(n^{1-1/D})$ of the X_i.

Hence the total number of X_i that can be fully or partially contained within a ball of radius R is upper bounded by

$$|B_R(y)| = O(n^{1-\alpha} + n^{1-1/D}) = O(n^{1-\min\{\alpha,1/D\}}) \ . \tag{31}$$

■

Supplementary Note 3 — Gadget characterisation

In this section, we give the proofs of Theorems 6 and 7, showing the equivalence of our notions of gadgets. The former is quite simple, but the latter requires several preparatory lemmas. In particular, we will make heavy use of the direct rotation — for a detailed introduction see the review of Bravyi et al.[3]. We summarise the basic definitions and properties here without proof.

Theorem 6 (Restatement of Theorem 16 in the Main Text)**.** *Suppose that $(H', \mathcal{A})$ is a (η,ϵ)-gadget for H. Then $(H', \mathcal{A})$ satisfies the (ζ,ϵ)-gadget property for H, where $\zeta = O(\eta)$.*

Theorem 7 (Restatement of Theorem 17 in the Main Text)**.** *Suppose that $(H', \mathcal{A})$ satisfies the (ζ,ϵ)-gadget property for H, where H, H', and P' act on $O(1)$ sites in $\mathcal{H} = \otimes_{i=1}^n \mathcal{H}_i$. Then $(H', \mathcal{A})$ is a (η,ϵ')-gadget for H, where $\eta = O(\epsilon) + O(\zeta^{\frac{1}{2}})$ and $\epsilon' = O(\epsilon) + O(\zeta)$.* *(Proof on page 9.)*

3.1 The direct rotation

Consider two states $|\psi\rangle, |\phi\rangle$ lying in some Hilbert space $\mathcal{H} \cong \mathbb{C}^N$. There are many unitary matrices $U \in \mathrm{U}(\mathcal{H})$ which rotate between these states (that is, $U|\psi\rangle = |\phi\rangle$), but a particularly natural choice is the unitary $U_{\psi\to\phi}$ which rotates only within the subspace spanned by $|\psi\rangle$ and $|\phi\rangle$. Defining the reflections $R_\psi = \mathrm{I} - 2|\psi\rangle\langle\psi|$ and $R_\phi = \mathrm{I} - 2|\phi\rangle\langle\phi|$, we can write

$$U_{\psi\to\phi} = \sqrt{R_\phi R_\psi} \ , \tag{32}$$

assuming it is well-defined. This is the direct rotation from $|\psi\rangle$ to $|\phi\rangle$.

This construction can be generalised to rotations between subspaces:

Definition 8 (Direct rotation)**.** Let $\mathcal{P}$ and $\mathcal{Q}$ be linear subspaces of equal dimension corresponding to

orthogonal projectors P and Q respectively. Define

$$R_{\mathcal{P}} = \mathrm{I} - 2P \ , \quad R_{\mathcal{Q}} = \mathrm{I} - 2Q \ , \tag{33}$$

then direct rotation between $\mathcal{P}$ and $\mathcal{Q}$ is

$$U_{\mathcal{P}\to\mathcal{Q}} = \sqrt{R_{\mathcal{Q}} R_{\mathcal{P}}} \ , \tag{34}$$

where the square root is taken with a branch cut along the negative axis and such that $\sqrt{1} = 1$. This is well-defined whenever $\|P - Q\| < 1$.

Then $U_{\mathcal{P}\to\mathcal{Q}}$ satisfies

$$U_{\mathcal{P}\to\mathcal{Q}} P U^{\dagger}_{\mathcal{P}\to\mathcal{Q}} = Q \ . \tag{35}$$

Moreover, as described by Bravyi et al.[3], the direct rotation may be written in terms of its generator: an anti-Hermitian operator $S = -S^{\dagger}$ which can be chosen so that $U_{\mathcal{P}\to\mathcal{Q}} = e^{S}$, with $\|S\| < \pi/2$ and which is off-diagonal with respect to both P and Q:

$$PSP = (\mathrm{I} - P)S(\mathrm{I} - P) = QSQ = (\mathrm{I} - Q)S(\mathrm{I} - Q) = 0 \ . \tag{36}$$

Notice that, writing $S = i\,\mathrm{diag}(\theta_1, \theta_2, \ldots, \theta_n)$ for $\theta_j \in (-\pi/2, \pi/2)$, we have

$$\|U_{\mathcal{P}\to\mathcal{Q}} - \mathrm{I}\,\| = \max_j |2\sin(\theta_j/2)| \ , \quad \|S\| = \max_j |\theta_j| \ , \tag{37}$$

and hence

$$\|S\| \leq \frac{\pi}{2\sqrt{2}} \|U_{\mathcal{P}\to\mathcal{Q}} - \mathrm{I}\,\| \ . \tag{38}$$

3.2 The gadget property from gadgets

Proof of Theorem 6. This follows directly from the definition, since for any $H_{\mathrm{else}} \in \mathrm{Herm}(\mathcal{H})$, we have

$$\begin{aligned} &\|P'(H' + H_{\mathrm{else}} \otimes \mathrm{I})P' - U\big((H + H_{\mathrm{else}}) \otimes P\big)U^{\dagger}\| \\ &\quad \leq \|P'H'P' - U(H \otimes P)U^{\dagger}\| + \|P'(H_{\mathrm{else}} \otimes \mathrm{I})P' - U(H_{\mathrm{else}} \otimes P)U^{\dagger}\| \ . \end{aligned} \tag{39}$$

The first term is bounded by ϵ by definition, and the second term can be bounded using

$$\begin{aligned} &\|P'(H_{\mathrm{else}} \otimes \mathrm{I})P' - U(H_{\mathrm{else}} \otimes P)U^{\dagger}\| \\ &\quad = \|(\mathrm{I} \otimes P)U^{\dagger}(H_{\mathrm{else}} \otimes \mathrm{I})U(\mathrm{I} \otimes P) - H_{\mathrm{else}} \otimes P\| \\ &\quad \leq 2\eta\|H_{\mathrm{else}}\| \ . \end{aligned} \tag{40}$$

Hence the gadget property is satisfied, putting $\tilde{P} = P$ and $\tilde{U}_{H_{\mathrm{else}}} := U$ for all H_{else}. ■

3.3 Gadgets from the gadget property

The proof of Theorem 7 requires Lemma 9, a basic linear algebra fact which we prove here for convenience. Two projectors P and Q commute if and only if they are simultaneously diagonalisable, in which case PQP is also a projector. This lemma says that this is also true in the approximate setting: $[P, Q]$ is small if and only if PQP is close to some projector $\tilde{P}$.

Lemma 9. *Let $P, Q \in \mathrm{Proj}(\mathcal{H})$ be projectors, and define*

$$f(P,Q) := \min_{\tilde{P} \in \mathrm{Proj}(\mathcal{H})} \|PQP - \tilde{P}\| \ . \tag{41}$$

Then

$$\|[P,Q]\| = \sqrt{f(P,Q) - f(P,Q)^2} \ . \tag{42}$$

Proof of Lemma 9. Write P and Q in the block-diagonal basis of P, so that

$$P = \begin{pmatrix} \mathrm{I} & 0 \\ 0 & 0 \end{pmatrix} , \quad Q = \begin{pmatrix} A & B \\ B^\dagger & C \end{pmatrix} , \tag{43}$$

for some matrices A, B, C. The requirement $Q \in \mathrm{Proj}(\mathcal{H})$ implies that $BB^\dagger = A(\mathrm{I} - A)$. We have

$$PQP = \begin{pmatrix} A & 0 \\ 0 & 0 \end{pmatrix} \ . \tag{44}$$

Let $\{\lambda_j\}_j$ be the eigenvalues of A; notice that these satisfy $0 \le \lambda_j \le 1$, since $0 \le PQP \le Q$. Then $f(P,Q)$ is given by

$$f(P,Q) = \max_j \big(\min\{|\lambda_j|, |1-\lambda_j|\} \big) \ . \tag{45}$$

To see why (45) holds, note that the upper bound on f follows by constructing $\tilde{P}$ to have the same eigenvectors as PQP, but with each eigenvalue replaced by either 0 or 1 depending on which is closer. The lower bound follows from Lemma 4.

Now we can compute

$$-[P,Q]^2 = \begin{pmatrix} BB^\dagger & 0 \\ 0 & B^\dagger B \end{pmatrix} , \tag{46}$$

hence

$$\|[P,Q]\|^2 = \|-[P,Q]^2\| = \|BB^\dagger\| = \|A(\mathrm{I} - A)\| = \max_j |\lambda_j||1-\lambda_j| \ . \tag{47}$$

Note that the maximising j in (45) and (47) must be the same (the functions $\min\{|\lambda|, |1-\lambda|\}$ and $|\lambda||1-\lambda|$ are both maximised by the λ_j closest to $1/2$), hence we can deduce

$$\|[P,Q]\|^2 = f(P,Q)\big(1 - f(P,Q)\big) \ , \tag{48}$$

which gives the result. ∎

In order to obtain the correct unitary U in the gadget definition, the proof of Theorem 7 requires constructing rotations between eigenspaces of different operators. The Davis-Kahan $\sin\theta$ theorem below provides a bound on the size of these rotations. This is also used in the proof of Proposition 17.

Lemma 10 (Davis-Kahan $\sin\theta$ theorem[4]). *Let $A, B \in \mathrm{Herm}(\mathcal{H})$, and take $P_A, P_B \in \mathrm{Proj}(\mathcal{H})$ projectors*

of equal rank which block-diagonalise A and B respectively, so that

$$A = P_A A P_A + P_A^\perp A P_A^\perp \ , \quad B = P_B B P_B + P_B^\perp B P_B^\perp \ . \tag{49}$$

Assume $\alpha, \beta \in \mathbb{R}$ and λ_{gap} are such that

$$\mathrm{spec}\left(A|_{P_A\mathcal{H}}\right) \subset [\alpha, \beta] \ , \quad \mathrm{spec}\left(B|_{P_B^\perp\mathcal{H}}\right) \subset \mathbb{R} \setminus (\alpha - \lambda_{\mathrm{gap}}, \beta + \lambda_{\mathrm{gap}}) \ . \tag{50}$$

Then the direct rotation $U \in \mathrm{U}(\mathcal{H})$ from P_A to P_B satisfies

$$\|U - \mathrm{I}\,\| \leq \frac{\sqrt{2}}{\lambda_{\mathrm{gap}}} \|(B - A)P_A\| \ . \tag{51}$$

Proof of Lemma 10. The statement of Davis et al.[4] is phrased in terms of a matrix $\Theta_0 = \mathrm{diag}(\theta_1, \theta_2, \ldots, \theta_n)$, where the eigenvalues (possibly excluding some 1's) of U are given by $e^{i\theta_j}$ and $\pi/2 \geq \theta_1 \geq \theta_2 \geq \cdots \geq \theta_n$. Specifically, the authors give the following result:

$$\lambda_{\mathrm{gap}} \|\sin\Theta_0\| \leq \|(B - A)P_A\| \ . \tag{52}$$

To recover our restatement of the theorem, we use the identity $|1 - e^{i\theta}| = |2\sin(\theta/2)|$ to deduce that

$$\|U - \mathrm{I}\,\| = |2\sin(\theta_1/2)| \leq \sqrt{2}|\sin\theta_1| = \sqrt{2}\|\sin\Theta_0\| \ . \tag{53}$$

■

The following lemmas from Bravyi et al.[5] are used extensively in the rest of the gadget proofs. They provide bounds on a series expansion of $e^S H e^{-S}$, for S small and anti-Hermitian, in particular showing that

$$e^S H e^{-S} = H + [S, H] + \frac{1}{2!}[S, [S, H]] + \frac{1}{3!}[S, [S, [S, H]]] + \ldots \ . \tag{54}$$

Lemma 11 (Bravyi et al., Lemma 1[5])**.** *Let S be an anti-Hermitian operator. Define a superoperator ad_S such that $\mathrm{ad}_S(X) = [S, X]$, and let ad_S^k be the k-fold composition of ad_S, with $\mathrm{ad}_S^0(X) = X$. For any operator H define $r_0(H) = \|e^S H e^{-S}\| = \|H\|$, $r_1(H) = \|e^S H e^{-S} - H\|$, and*

$$r_k(H) = \|e^S H e^{-S} - \sum_{p=0}^{k-1} \frac{1}{p!} \mathrm{ad}_S^p(H)\| \ , \quad k \geq 2 \ . \tag{55}$$

Then for all $k \geq 0$ one has

$$r_k(H) \leq \frac{1}{k!} \|\,\mathrm{ad}_S^k(H)\| \ . \tag{56}$$

Lemma 12 (Bravyi et al., Lemma 2[5])**.** *Let $S = \sum_i S_i$ and $H = \sum_j H_j$ be any $O(1)$-local operators acting on n qubits with interaction strengths J_S and J_H respectively (i.e. $\|S_i\| \leq J_S$ and $\|H_j\| \leq J_H$ for all i and j). Let each qubit be acted on non-trivially by $O(1)$ terms in both S and H. Then, for any $k = O(1)$,*

$$\|\,\mathrm{ad}_S^k(H)\| = O(nJ_S^k J_H) \ . \tag{57}$$

These lemmas provide us with the necessary tools to prove Theorem 7.

Proof of Theorem 7. The idea of the proof is as follows.

By the gadget property, we have

$$\|P'(H' + H_{\text{else}} \otimes \mathrm{I})P' - \tilde{U}_{H_{\text{else}}}\big((H + H_{\text{else}}) \otimes \tilde{P}\big)\tilde{U}^{\dagger}_{H_{\text{else}}}\| \leq \epsilon + \zeta\|H_{\text{else}}\| , \tag{58}$$

for any $H_{\text{else}} \in \mathrm{Herm}(\mathcal{H})$.

1. First we consider (58) the case where H_{else} dominates the expression, and argue that that P' is "almost" a projector $\mathrm{I} \otimes P$ on $\mathcal{A}$.
2. Next, by setting $H_{\text{else}} = 0$ in (58), we observe that $P'H'P'$ has approximately the same spectrum as $H \otimes P$.
3. By setting $H_{\text{else}} = -H$ in (58), we argue that $P'H'P' \approx H \otimes P$.
4. Using steps 2 and 3, and Lemma 10, we construct a rotation U such that $\|P'H'P' - U(H \otimes P)U^{\dagger}\| \leq \epsilon$, by inductively rotating each eigenspace.

Here we start step 1. Assume that $\|H_{\text{else}}\| = 1$, and let $\lambda > 0$. Then putting $H_{\text{else}} \mapsto \lambda H_{\text{else}}$ in (58) yields

$$\|P'(H_{\text{else}} \otimes \mathrm{I})P' - \tilde{U}_{\lambda H_{\text{else}}}(H_{\text{else}} \otimes \tilde{P})\tilde{U}^{\dagger}_{\lambda H_{\text{else}}}\| \leq \zeta + O(\lambda^{-1}) , \tag{59}$$

for large λ, which in particular, by Lemma 4, implies that $P'(H_{\text{else}} \otimes \mathrm{I})P'$ has the same spectrum as $H_{\text{else}} \otimes \tilde{P}$, up to error ζ. (That is, the kth smallest eigenvalue, counted with multiplicity, of $P'(H_{\text{else}} \otimes \mathrm{I})P'$, differs from that of $H_{\text{else}} \otimes \tilde{P}$ by an absolute error of at most ζ.)

In particular, if $H_{\text{else}} = Q \in \mathrm{Proj}(\mathcal{H})$ is a projection, then so is $P'(Q \otimes \mathrm{I})P'$ (up to spectral error ζ). Hence by Lemma 9, we have

$$\|[P', Q \otimes \mathrm{I}]\| \leq \sqrt{\zeta} . \tag{60}$$

Without loss of generality we may assume that $\dim \mathcal{H} = K = O(1)$, by disregarding all the systems on which H, H', and P' do not act, using the assumptions of the theorem. So by writing H_{else} as a linear combination of at most K projections, we have

$$\|[P', H_{\text{else}} \otimes \mathrm{I}]\| \leq \pi K \sqrt{\zeta} , \tag{61}$$

for all $H_{\text{else}} \in \mathrm{Herm}(\mathcal{H})$ with $\|H_{\text{else}}\| \leq \pi$.

Therefore, for any $V \in \mathrm{U}(\mathcal{H})$, we can write $V = e^{iH_{\text{else}}}$ for some H_{else} as above, and then by Lemma 11,

$$\|(V \otimes \mathrm{I})P'(V^{\dagger} \otimes \mathrm{I}) - P'\| \leq \pi K \sqrt{\zeta} . \tag{62}$$

Integrating over all $V \in \mathrm{U}(\mathcal{H})$ using the Haar measure (normalised with $\int \mathrm{d}\,V = 1$) yields

$$\left\| \int \mathrm{d}\,V (V \otimes \mathrm{I})P'(V^{\dagger} \otimes \mathrm{I}) - P' \right\| \leq \pi K \sqrt{\zeta} , \tag{63}$$

but

$$\int \mathrm{d}\,V (V \otimes \mathrm{I})P'(V^{\dagger} \otimes \mathrm{I}) = K^{-1}\, \mathrm{I}_{\mathcal{H}} \otimes \mathrm{tr}_{\mathcal{H}}[P'] , \tag{64}$$

hence

$$\|P' - \mathrm{I}_{\mathcal{H}} \otimes K^{-1}\, \mathrm{tr}_{\mathcal{H}}[P']\| \leq \pi K \sqrt{\zeta} . \tag{65}$$

In particular, by Lemma 4 this implies that $K^{-1}\operatorname{tr}_{\mathcal{H}}[P']$ has spectrum in $[-\pi K\sqrt{\zeta}, \pi K\sqrt{\zeta}] \cup [1-\pi K\sqrt{\zeta}, 1+\pi K\sqrt{\zeta}]$ (where for sufficiently small ζ there will be a gap). We can therefore construct a projector $P \in \mathrm{Proj}(\mathcal{A})$ by rounding the eigenvalues of $K^{-1}\operatorname{tr}_{\mathcal{H}}[P']$ to the nearest integer, which satisfies

$$\|P' - \mathrm{I}\otimes P\| \leq 2\pi K\sqrt{\zeta}\ . \tag{66}$$

Now we can apply Lemma 10, using $A = P_A = P'$, $B = P_B = \mathrm{I}\otimes P$, and $\lambda_{\text{gap}} = 1$. Then for ζ small enough, the direct rotation W from $\mathrm{I}\otimes P$ to P' satisfies

$$P' = W(\mathrm{I}\otimes P)W^\dagger\ , \quad \|W - \mathrm{I}\| \leq 2\sqrt{2}\pi K\sqrt{\zeta}\ , \tag{67}$$

which completes step 1. Without loss of generality we can now adjust the $\tilde{U}_{H_{\text{else}}}$ so that $\tilde{P} = P$, since (59) holds for all H_{else} and therefore $\operatorname{rank} P' = \operatorname{rank} \mathrm{I}\otimes P = \operatorname{rank} \mathrm{I}\otimes\tilde{P}$ for small enough ζ. Hence for all $H_{\text{else}} \in \mathrm{Herm}(\mathcal{H})$ we have

$$\|P'(H' + H_{\text{else}}\otimes \mathrm{I})P' - \tilde{U}_{H_{\text{else}}}\big((H + H_{\text{else}})\otimes P\big)\tilde{U}^\dagger_{H_{\text{else}}}\| \leq \epsilon + \zeta\|H_{\text{else}}\|\ . \tag{68}$$

With the above expression, we can begin steps 2 and 3. Putting $H_{\text{else}} = 0$, this becomes

$$\|P'H'P' - \tilde{U}(0)(H\otimes P)\tilde{U}^\dagger(0)\| \leq \epsilon\ , \tag{69}$$

and putting $H_{\text{else}} = -H$ we have

$$\|P'H'P' - P'(H\otimes \mathrm{I})P'\| \leq \epsilon + \zeta\|H\|\ . \tag{70}$$

Moreover, by (67) we can bound

$$\begin{aligned}
\|P'(H\otimes \mathrm{I})P' - H\otimes P\| &= \|W(\mathrm{I}\otimes P)W^\dagger(H\otimes \mathrm{I})W(\mathrm{I}\otimes P)W^\dagger - H\otimes P\| \\
&= \|(W-\mathrm{I})(\mathrm{I}\otimes P)W^\dagger(H\otimes \mathrm{I})W(\mathrm{I}\otimes P)W^\dagger \\
&\quad + (\mathrm{I}\otimes P)(W^\dagger - \mathrm{I})(H\otimes \mathrm{I})W(\mathrm{I}\otimes P)W^\dagger \\
&\quad + (H\otimes P)(W-\mathrm{I})(\mathrm{I}\otimes P)W^\dagger \\
&\quad + (H\otimes P)(W^\dagger - \mathrm{I})\| \\
&\leq 4\|H\|\cdot\|W-\mathrm{I}\| \\
&\leq 8\sqrt{2}\pi K\sqrt{\zeta}\|H\|\ .
\end{aligned} \tag{71}$$

Combining (70) and (71), we complete step 3:

$$\|P'H'P' - H\otimes P\| \leq \epsilon + (\zeta + 8\sqrt{2}\pi K\sqrt{\zeta})\|H\| := \delta\ . \tag{72}$$

Now we begin step 4. Let $H^{(0)} = H\otimes P$. Write the eigenvalues of this operator as $\{\lambda_k\}_{k=1}^{M+1}$, where $\lambda_1 = 0$ and $0 < \lambda_2 < \cdots < \lambda_{M+1}$ are the M distinct eigenvalues of H. If H has any non-positive eigenvalues, then we shift both H and H' by a $O(1)$ factor of the identity for the duration of the proof; notice that the gadget property then still holds up to a redefined ϵ which does not affect the conclusions of this theorem. To see this, note that for $\mu \in \mathbb{R}$,

$$\begin{aligned}
\|P'(H' + \mu\,\mathrm{I} + H_{\text{else}}\otimes \mathrm{I})P' - \tilde{U}_{H_{\text{else}}}\big((H + \mu\,\mathrm{I} + H_{\text{else}})\otimes\tilde{P}\big)\tilde{U}^\dagger_{H_{\text{else}}}\| &\leq \epsilon + \zeta\|\mu\,\mathrm{I} + H_{\text{else}}\| \\
&\leq (\epsilon + \mu\zeta) + \zeta\|H_{\text{else}}\|\ ,
\end{aligned} \tag{73}$$

using (58) and absorbing $\mu\,\mathrm{I}$ into H_{else}. Hence $H' \mapsto H' + \mu\,\mathrm{I}$ and $H \mapsto H + \mu\,\mathrm{I}$ also satisfy the

assumptions of the theorem, up to replacing $\epsilon \mapsto \epsilon + \mu\zeta$. Moreover, if we can show that $H' + \mu\,\mathrm{I}$ is an (ϵ, η)-gadget for $H + \mu\,\mathrm{I}$, then applying the gadget definition shows that

$$\begin{aligned} \|P'H'P' - U(H \otimes P)U^\dagger\| &= \|P'(H' + \mu\,\mathrm{I})P' - U\big((H + \mu\,\mathrm{I}) \otimes P\big)U^\dagger\| \\ &\leq \epsilon \ , \end{aligned} \tag{74}$$

hence H' is an (ϵ, η)-gadget for H.

We define

$$\lambda_{\text{gap}} = \min_{j \neq k} |\lambda_j - \lambda_k| \ , \tag{75}$$

which is $O(1)$ since H acts on $O(1)$ sites, and does not scale with η or ϵ. Let $\mathcal{P}_k$ be the eigenspace of $H^{(0)}$ corresponding to λ_k.

We also diagonalise $P'H'P'$ — see Supplementary Figure 1. By (69) and Weyl's inequality we can write the eigenvalues as $\{\mu_k^{(i_k)}\}$ such that

$$|\mu_k^{(i_k)} - \lambda_k| \leq \epsilon \ , \quad \text{for all } i_k\text{, and for all } k. \tag{76}$$

Let $\mathcal{P}'_k$ be the eigenspace of $P'H'P'$ corresponding to the eigenvalues $\{\mu_k^{(i_k)}\}_{i_k}$, which by (69) satisfies $\dim \mathcal{P}'_k = \dim \mathcal{P}_k$ for ϵ sufficiently small. Note that for $j \neq k$ we have

$$|\mu_j^{(i_j)} - \lambda_k| \geq \lambda_{\text{gap}} - \epsilon \ . \tag{77}$$

We aim to construct a unitary operator which rotates all of the $\mathcal{P}_i$ onto the $\mathcal{P}'_i$ eigenspaces. We do this by induction, defining $W^{(k)}$ to be a unitary operator which performs these rotations for $i = 1, \ldots, k$. Moreover the define $H^{(k)} = W^{(k)} H^{(0)} (W^{(k)})^\dagger$ to be the version of $H \otimes P$ whose first k eigenspaces have been rotated in this way. We will use bounds on the direct rotation provided by Lemma 10; we will see that the direct rotations are well-defined for sufficiently small ζ and ϵ.

The inductive construction we use will bound the rotations by $\|W^{(k)} - \mathrm{I}\,\| \leq \omega_k$, where

$$\omega_k = \frac{\delta}{2\|H\|} \left(\left[1 + \frac{2\sqrt{2}\|H\|}{\lambda_{\text{gap}} - \epsilon} \right]^k - 1 \right) \ . \tag{78}$$

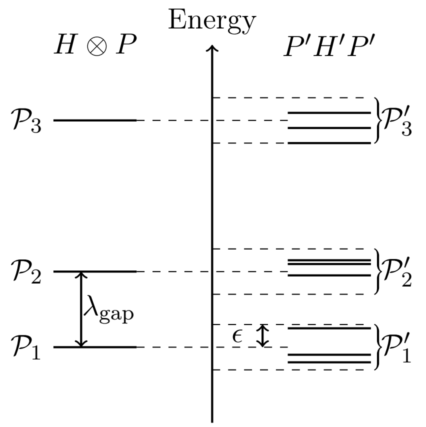

Supplementary Figure 1: The degenerate eigenvalues of $H \otimes P$ are approximated by eigenvalues of $P'H'P'$ up to error ϵ, corresponding to eigenspaces $\mathcal{P}'_i$. We construct a unitary operator which rotates the eigenspaces $\mathcal{P}_i$ onto the $\mathcal{P}'_i$

We now inductively define the $H^{(k)} \in \mathrm{Herm}(\mathcal{H} \otimes \mathcal{A})$ and $W^{(k)} \in \mathrm{U}(\mathcal{H} \otimes \mathcal{A})$ as described above. For the base case, we see that clearly $H^{(0)}$ satisfies the conditions with the trivial $W^{(0)} = \mathrm{I}$.

For the inductive step, suppose we are given $H^{(k-1)}$ and $W^{(k-1)}$. Notice that

$$\|P'H'P' - H^{(k-1)}\| \leq \delta + 2\omega_{k-1}\|H\| \ , \tag{79}$$

using (72) and the bound $\|W^{(k-1)} - \mathrm{I}\,\| \leq \omega_{k-1}$. Hence, applying Lemma 10 to $(P'H'P')|_{\oplus_{j\geq k}\mathcal{P}'_j}$, and $H^{(k-1)}|_{\oplus_{j\geq k}\mathcal{P}'_j}$ (using the fact that $H^{(k-1)}$ is constructed to leave $\oplus_{j\leq k-1}\mathcal{P}'_j$, and hence also $\oplus_{j\geq k}\mathcal{P}'_j$, invariant), we may construct the direct rotation $V^{(k)}$ on $\oplus_{j\geq k}\mathcal{P}'_j$ which maps from $W^{(k-1)}\mathcal{P}_k(W^{(k-1)})^\dagger$ to $\mathcal{P}'_k$, and which satisfies

$$\begin{aligned}\|V^{(k)} - \mathrm{I}\,\| &\leq \frac{\sqrt{2}}{\lambda_{\text{gap}} - \epsilon}\|(P'H'P')|_{\oplus_{j\geq k}\mathcal{P}'_j} - (H^{(k-1)})|_{\oplus_{j\geq k}\mathcal{P}'_j}\| \\ &\leq \frac{\sqrt{2}}{\lambda_{\text{gap}} - \epsilon}(\delta + 2\omega_{k-1}\|H\|) \ .\end{aligned} \tag{80}$$

For the above step, it is necessary to verify that the direct rotation is well-defined. If it were not, then there would be a nonzero vector $|\psi\rangle \in W^{(k-1)}\mathcal{P}(W^{(k-1)})^\dagger \cap (\mathcal{P}'_k)^\perp$. Then, by the definition of these subspaces,

$$\langle\psi|P'H'P'|\psi\rangle \geq \lambda_{k+1} - \epsilon \geq \lambda_k + (\lambda_{\text{gap}} - \epsilon) \ , \quad \langle\psi|H^{(k-1)}|\psi\rangle = \lambda_k \ , \tag{81}$$

which would imply that

$$\|P'H'P' - H^{(k-1)}\| \geq \lambda_{\text{gap}} - \epsilon \ . \tag{82}$$

By (79), this is prohibited for sufficiently small ζ and ϵ — so we can safely assume that the direct rotation is well-defined.

Now we can let

$$W^{(k)} = (\mathrm{I}_{\oplus_{j<k}\mathcal{P}'_j} \oplus V^{(k)})W^{(k-1)} \ , \tag{83}$$

and

$$H^{(k)} = W^{(k)}H^{(0)}(W^{(k)})^\dagger \ , \tag{84}$$

which satisfies

$$\|W^{(k)} - \mathrm{I}\,\| \le \omega_{k-1} + \frac{\sqrt{2}}{\lambda_{\text{gap}} - \epsilon}(\delta + 2\omega_{k-1}\|H\|) = \omega_k \ . \tag{85}$$

After $M+1$ inductive steps, we have constructed the operator

$$H^{(M+1)} = W^{(M+1)}(H \otimes P)(W^{(M+1)})^\dagger \ , \tag{86}$$

whose λ_k-eigenspace is $\mathcal{P}'_k$ for all k. Hence

$$\|P'H'P' - W^{(M+1)}(H \otimes P)(W^{(M+1)})^\dagger\| \le \epsilon \ . \tag{87}$$

Moreover, since H has no zero eigenvalues (since otherwise we shifted by a factor of the identity), we are guaranteed that the null space of $H \otimes P$ is exactly that of $(\mathrm{I} \otimes P)$, and $\mathrm{rank}(H \otimes P) = \mathrm{rank}(\mathrm{I} \otimes P)$. By (67) and (69) we know that $\mathrm{rank}(P') = \mathrm{rank}(\mathrm{I} \otimes P)$ and $\mathrm{rank}(P'H'P') = \mathrm{rank}(H \otimes P)$ for sufficiently small ζ and ϵ. So

$$\mathrm{rank}(P') = \mathrm{rank}(\mathrm{I} \otimes P) = \mathrm{rank}(H \otimes P) = \mathrm{rank}(P'H'P') \ , \tag{88}$$

and the null space of $P'H'P'$ is exactly that of P'. Hence by construction of $W^{(M+1)}$,

$$P' = W^{(M+1)}(\mathrm{I} \otimes P)(W^{(M+1)})^\dagger \ . \tag{89}$$

We have therefore shown that $(H', \mathcal{A})$ is a gadget as in our definition, using $U = W^{(M+1)}$, with accuracy ϵ (possibly with an additional $O(\zeta)$ if shifting of H' was necessary earlier in the proof) and $\eta = O(\epsilon) + O(\sqrt{\zeta})$ given explicitly by

$$\eta = \omega_{M+1} = \frac{1}{2\|H\|}\left[\left(\frac{2\sqrt{2}}{\lambda_{\text{gap}} - \epsilon}\|H\| + 1\right)^M - 1\right]\left[\epsilon + (\zeta + 8\sqrt{2}\pi K\sqrt{\zeta})\|H\|\right] \ . \tag{90}$$

■

Supplementary Note 4 — Gadget combination

In this section we prove the various gadget combination results. Below is a restatement of the general setup for the section.

Setup 13 (Restatement of Setup 22 in the Main Text)**.** *Let $H \in \mathrm{Herm}(\mathcal{H})$ be a Hamiltonian on n sites, $\mathcal{H} = \otimes_{i=1}^n \mathcal{H}_i$. Assume $H = \sum_{i=1}^N H_i$, where $N = O(n)$, such that each H_i acts on at most $k = O(1)$ of the sites $\mathcal{H}_i$, and each site participates in at most $d = O(1)$ interactions. Assume also that H has bounded interaction strengths, that is, $\|H_i\| \le J$ for all i.*

In the below propositions we consider a family (depending on n) of gadgets $(H'_i, \mathcal{A}_i)$ for H_i, with U_i, P_i, and P'_i defined as in the gadget definition, for each i. Assume that $\mathcal{A}_i$ consists of $O(1)$ ancillary sites and that H'_i is a local Hamiltonian consisting of $O(1)$ interactions, such that

$$\|H'_i\| \le J' \ , \quad \|(\mathrm{I} \otimes P_i)H'_i(\mathrm{I} \otimes P_i^\perp)\| \le J'_O \ . \tag{91}$$

4.1 Combination of general gadgets

Proposition 14 (Restatement of Proposition 23 in the Main Text)**.** *Let $H = \sum_i H_i$ be as in Setup 13, and suppose that each $(H'_i, \mathcal{A}_i)$ defines a (η, ϵ)-gadget for H_i.*

Define

$$H' = \sum_i H'_i \in \mathrm{Herm}\left(\mathcal{H} \otimes (\otimes_i \mathcal{A}_i)\right) \ . \tag{92}$$

Then $(H', \otimes_i \mathcal{A}_i)$ *is a* (η', ϵ')*-gadget for* H*, where*

$$\epsilon' = O(n\epsilon + n\eta J + n\eta^3 J'_O + n\eta^4 J') \ , \quad \eta' = O(n\eta) \ . \tag{93}$$

Here we introduce some preparatory lemmas before proving Proposition 14.

The first lemma allows us to immediately reduce gadgets to the case that the unitary U is a direct rotation, defined in Definition 8. This allows us to write $U = e^S$ for S the generator of the direct rotation — the off-diagonal properties of S will simplify calculations considerably.

Lemma 15. *Suppose* $(H', \mathcal{A})$ *is a* (η, ϵ)*-gadget for* H*, where* $\eta < \sqrt{2}$*. Let* $\tilde{\epsilon} = \epsilon + 4\eta\|H\|$*.*

Then $(H', \mathcal{A})$ *is also a* $(\eta, \tilde{\epsilon})$*-gadget for* H*, where the unitary* U *in the definition can be assumed to be the direct rotation* W[3] *between the subspaces defined by* $(\mathrm{I} \otimes P)$ *and* P'*.*

Proof of Lemma 15. By the gadget definition we have U such that

$$P' = U(\mathrm{I} \otimes P)U^\dagger \ , \quad \|U - \mathrm{I}\| \leq \eta \ . \tag{94}$$

As shown by Davis et al.[6], the direct rotation W between $(\mathrm{I} \otimes P)$ and P' minimises $\|W - \mathrm{I}\|$ subject to the first equality above, hence we have

$$P' = W(\mathrm{I} \otimes P)W^\dagger \ , \quad \|W - \mathrm{I}\| \leq \eta \ . \tag{95}$$

So

$$\begin{aligned} \|P'H'P' - W(H \otimes P)W^\dagger\| &\leq \epsilon + \|U(H \otimes P)U^\dagger - W(H \otimes P)W^\dagger\| \\ &\leq \epsilon + \|U(H \otimes P)U^\dagger - H \otimes P\| \\ &\quad + \|W(H \otimes P)W^\dagger - H \otimes P\| \\ &\leq \epsilon + 4\eta\|H\| \ . \end{aligned} \tag{96}$$

∎

A gadget H' for H has $\|P'H'P'\| \leq \|H\| + \epsilon$ by definition, however outside the span of P' there are no bounds on H'. For η small, P' will be close to the projector $\mathrm{I} \otimes P$. The following lemma provides a bound for H' when instead restricted to the span of $\mathrm{I} \otimes P$.

Lemma 16. *Suppose* $(H', \mathcal{A})$ *is a* (η, ϵ)*-gadget for* H*, with* U*,* P'*, and* P *as in the definition, and where* U *is the direct rotation between* $(\mathrm{I} \otimes P)$ *and* P'*. Assume* $\|H'\| \leq J'$*, and*

$$\|(\mathrm{I} \otimes P)H'(\mathrm{I} \otimes P^\perp)\| \leq J'_O \ . \tag{97}$$

Then

$$\|(\mathrm{I} \otimes P)H'(\mathrm{I} \otimes P)\| \leq \|H\| + O(\epsilon + \eta J'_O + \eta^2 J') \ . \tag{98}$$

Proof of Lemma 16. Let S be the generator of the direct rotation U, so that $\|S\| = O(\eta)$ (see (38)) satisfies

$$P' = e^S (\mathrm{I} \otimes P) e^{-S} \ . \tag{99}$$

Hence

$$\|(\mathrm{I} \otimes P) H' (\mathrm{I} \otimes P)\| \le \|(\mathrm{I} \otimes P)(e^{-S} H' e^S - H')(\mathrm{I} \otimes P)\| + \|P' H' P'\| \ . \tag{100}$$

By the gadget definition, we have $\|P'H'P'\| \le \|H\| + \epsilon$, and using Lemma 11 we can bound the first term as

$$\|(\mathrm{I} \otimes P)(e^{-S} H' e^S - H')(\mathrm{I} \otimes P)\| \le \|(\mathrm{I} \otimes P)[S, H'](\mathrm{I} \otimes P)\| + O(\eta^2 J') \ . \tag{101}$$

Furthermore, since S is off-diagonal with respect to $(\mathrm{I} \otimes P)$ we have

$$\|(\mathrm{I} \otimes P)[S, H'](\mathrm{I} \otimes P)\| \le O(\eta J'_O) \ , \tag{102}$$

completing the proof. ■

We now have the necessary tools to prove the gadget combination result Proposition 14. We are provided with the gadgets $(H'_i, \mathcal{A}_i)$ for each of the H_i (corresponding to U_i, P_i, P'_i as in the gadget definition), which immediately suggest using $P = \otimes_i P_i$ for the gadget $H' = \sum_i H'_i$ for $H = \sum_i H_i$. It is not immediately clear what unitary U, and hence projector P', should be used here, since the U_i do not necessarily commute so cannot be naïvely composed. The direct rotation provides a natural choice, however; writing $U_i = e^{S_i}$ for all i, we can choose $U = e^{\sum_i S_i}$. The content of the proof is just a long computation to verify that this choice indeed satisfies the gadget definition.

The following proof is a generalisation of a result of Bravyi et al.[5], for which which similar techniques are used.

Proof of Proposition 14. We begin by reducing to the case of the direct rotation. By Lemma 15, we may replace ϵ with

$$\tilde{\epsilon} = \epsilon + 4\eta J = O(\epsilon + \eta J) \ , \tag{103}$$

and hence assume that each gadget $(H'_i, \mathcal{A}_i)$ uses the direct rotation $U_i = e^{S_i}$. Specifically, there exists $P_i \in \mathrm{Proj}(\mathcal{A}_i)$ such that

$$\|(\mathrm{I} \otimes P_i) e^{-S_i} H'_i e^{S_i} (\mathrm{I} \otimes P_i) - H_i \otimes P_i\| \le \tilde{\epsilon} \ , \tag{104}$$

where S_i is the generator of the direct rotation between the projectors $\mathrm{I} \otimes P_i$ and $P'_i := e^{S_i}(\mathrm{I} \otimes P_i) e^{-S_i}$. We have $\|S_i\| \le O(\eta)$ and S_i is an anti-Hermitian operator which is block off-diagonal with respect to the projectors $\mathrm{I} \otimes P_i$ and P'_i[3].

We define the operators

$$P := \otimes_i P_i \in \mathrm{Proj}(\otimes_i \mathcal{A}_i) \ , \quad S = \sum_i S_i \ . \tag{105}$$

We now use the triangle inequality along with Lemmas 11-12 to bound

$$\begin{aligned} &\|(\mathrm{I} \otimes P) e^{-S} H' e^S (\mathrm{I} \otimes P) - H \otimes P\| \\ &\quad \le \sum_i \|(\mathrm{I} \otimes P)(H'_i - [S, H'_i] + \frac{1}{2}[S, [S, H'_i]] - \frac{1}{6}[S, [S, [S, H'_i]]])(\mathrm{I} \otimes P) \\ &\quad - H_i \otimes P\| + O(n\eta^4 J') \ . \end{aligned} \tag{106}$$

Now we bound the terms in the norm separately, using that S_j is block-off-diagonal with respect to

$(\mathrm{I}\otimes P_j)$, and that $(\mathrm{I}\otimes P_j)$ commutes with H'_k and S_k if $j \neq k$ since P_j acts only on the ancillary $\mathcal{A}_j$ system, whilst H'_k and S_k act on $\mathcal{H}\otimes\mathcal{A}_k$.

- $(\mathrm{I}\otimes P)[S, H'_i](\mathrm{I}\otimes P)$:

  Expanding $S = \sum_j S_j$, notice that $(\mathrm{I}\otimes P)[S_j, H'_i](\mathrm{I}\otimes P) = 0$ whenever $j \neq i$, since then we can commute $(\mathrm{I}\otimes P_j)$ past H'_i. Hence

$$(\mathrm{I}\otimes P)[S, H'_i](\mathrm{I}\otimes P) = (\mathrm{I}\otimes P)[S_i, H'_i](\mathrm{I}\otimes P) \ . \tag{107}$$

- $(\mathrm{I}\otimes P)[S, [S, H'_i]](\mathrm{I}\otimes P)$:

  Note that if $j \neq k$, then

$$(\mathrm{I}\otimes P)[S_j, [S_k, H'_i]](\mathrm{I}\otimes P) = 0 \ , \tag{108}$$

  since at least one of them must be also different to i. Then, for instance if $j \neq i$, we can commute $(\mathrm{I}\otimes P_j)$ past H'_i and S_k. Hence it remains to consider terms of the form

$$(\mathrm{I}\otimes P)[S_j, [S_j, H'_i]](\mathrm{I}\otimes P) \ . \tag{109}$$

  In this case, if $j \neq i$, then we have

$$\begin{aligned}\|(\mathrm{I}\otimes P)[S_j, [S_j, H'_i]](\mathrm{I}\otimes P)\| &\leq 4\|S_j\|^2\|(\mathrm{I}\otimes P_i)H'_i(\mathrm{I}\otimes P_i)\| \\ &\leq O(\eta^2 J + \eta^3 J'_O + \eta^4 J') \ ,\end{aligned} \tag{110}$$

  using Lemma 16, and neglecting the $O(\eta^2\epsilon)$ term which is dominated by the other terms in the regime of small ϵ to which the proposition applies. Hence

$$(\mathrm{I}\otimes P)[S, [S, H'_i]](\mathrm{I}\otimes P) = (\mathrm{I}\otimes P)[S_i, [S_i, H'_i]](\mathrm{I}\otimes P) + O(\eta^2 J + \eta^3 J'_O + \eta^4 J') \ . \tag{111}$$

  Here we have used the assumptions of bounded locality and degree in Setup 13. This ensures that only $O(1)$ of the local terms in S appear in the commutator $[S, H'_i]$, and similarly for $[S, [S, H'_i]]$.

- $(\mathrm{I}\otimes P)[S, [S, [S, H'_i]]](\mathrm{I}\otimes P)$:

  Here we consider terms of the form $(\mathrm{I}\otimes P)[S_j, [S_k, [S_l, H'_i]]](\mathrm{I}\otimes P)$ in various situations. Firstly, note that if none of j, k, l are equal to i then we can commute $(\mathrm{I}\otimes P_i)$ into the commutator to obtain

$$\begin{aligned}\|(\mathrm{I}\otimes P)[S_j, [S_k, [S_l, H'_i]]](\mathrm{I}\otimes P)\| &\leq 8\|S_j\|\|S_k\|\|S_l\|\|(\mathrm{I}\otimes P_i)H'_i(\mathrm{I}\otimes P_i)\| \\ &\leq O(\eta^3 J + \eta^4 J'_O + \eta^5 J') \ ,\end{aligned} \tag{112}$$

  by Lemma 16, neglecting the $O(\eta^3\epsilon)$ term.

  If exactly two of the j, k, l are equal to i ($k = l = i \neq j$, say), then we can commute $(\mathrm{I}\otimes P_j)$ past the other terms to kill the S_j term, and the expression vanishes.

  If exactly one of the j, k, l is equal to i, then by commuting $(\mathrm{I}\otimes P_i)S_i = S_i(\mathrm{I}\otimes P_i^{\perp})$, we arrive at

$$\begin{aligned}\|(\mathrm{I}\otimes P)[S_j, [S_k, [S_l, H'_i]]](\mathrm{I}\otimes P)\| &\leq O(\eta^3)\|(\mathrm{I}\otimes P_i^{\perp})H'_i(\mathrm{I}\otimes P_i)\| \\ &= O(\eta^3 J'_O) \ .\end{aligned} \tag{113}$$

Hence

$$(\mathrm{I} \otimes P)[S,[S,[S,H_i']]](\mathrm{I} \otimes P) = (\mathrm{I} \otimes P)[S_i,[S_i,[S_i,H_i']]](\mathrm{I} \otimes P) + O(\eta^3 J + \eta^3 J_O' + \eta^5 J') \ , \tag{114}$$

once again using the locality assumptions of Setup 13.

So putting the above bounds together and applying Lemma 11, we obtain

$$\begin{aligned}\|(\mathrm{I} \otimes P)e^{-S}H'e^{S}(\mathrm{I} \otimes P) - H \otimes P\| &\leq \sum_i \|(\mathrm{I} \otimes P)e^{-S_i}H_i'e^{S_i}(\mathrm{I} \otimes P) - H_i \otimes P\| \\ &\quad + O(n\eta^2 J + n\eta^3 J_O' + n\eta^4 J') \\ &\leq O(n\epsilon + n\eta J + n\eta^3 J_O' + n\eta^4 J') \ ,\end{aligned} \tag{115}$$

where the last inequality follows because the H_i' are gadgets for the H_i.

Noting also that $\|e^S - \mathrm{I}\| = O(n\eta)$, this completes the proof that $(H', \mathcal{A})$ is a (η', ϵ')-gadget for H as required.

■

4.2 Combination of low-energy gadgets

Proposition 17 (Restatement of Proposition 24 in the Main Text)**.** *Let $H = \sum_i H_i$ be as in Setup 13, and suppose that each $(H_i', \mathcal{A}_i)$ defines a (Δ, η, ϵ)-gadget for H_i, where*

$$\Delta \geq \frac{\|H\| + J + N(\epsilon + 2J\eta)}{\frac{1}{4} - 2\eta} = O(nJ) \ , \tag{116}$$

and assume that the scaling of η with n is bounded as

$$\eta = o(n^{-\frac{1}{2}}) \ , \tag{117}$$

and moreover that, for large J',

$$n\epsilon + n\eta J + n\eta^3 J_O' + n\eta^4 J' = o(J') \ , \quad J' = O(\Delta) \ . \tag{118}$$

Define

$$H' = \sum_i H_i' \in \mathrm{Herm}(\mathcal{H} \otimes (\otimes_i \mathcal{A}_i)) \ . \tag{119}$$

Then $(H', \otimes_i \mathcal{A}_i)$ is a $(\Delta', \eta', \epsilon')$-gadget for H, where

$$\Delta' = \frac{1}{2}\Delta \ , \quad \epsilon' = O(n\epsilon + n\eta J + n\eta^3 J_O' + n^3\eta^4 J') \ , \quad \eta' = O(n\eta) \ . \tag{120}$$

Having proved Proposition 14, we know that the H' from Proposition 17 is an (η', ϵ')-gadget for H. It remains to prove that it is in fact a $(\Delta', \eta', \epsilon')$-gadget, which requires replacing the projector $\tilde{P} = e^S(\mathrm{I} \otimes P)e^{-S}$ in (115) by a low-energy projector $P_{\leq \Delta'(H')}$. This requires the use of the following corollary to the Davis-Kahan $\sin\theta$ theorem (Lemma 10).

Lemma 18. *Let $A \in \mathrm{Herm}(\mathcal{H})$, and let $P \in \mathrm{Proj}(\mathcal{H})$ be a projector of the same rank as $P_{\leq \Delta(A)}$, where $P_{\leq \Delta(A)} \in \mathrm{Proj}(\mathcal{H})$ is the projector onto the eigenvectors of A with eigenvalues less than Δ. Suppose that $\|PAP\| \leq \lambda$.*

Then, for any $\Delta > \lambda$, the direct rotation $U \in \mathrm{U}(\mathcal{H})$ from P to $P_{\leq \Delta(A)}$ satisfies

$$\|U - \mathrm{I}\| \leq \frac{\sqrt{2}}{\Delta - \lambda} \|P^{\perp} A P\| \ . \tag{121}$$

Proof of Lemma 18. Follows from Lemma 10 using $A \mapsto PAP$, $B \mapsto A$. ∎

Proof of Proposition 17. By Proposition 14, we have

$$\tau := \|(\mathrm{I} \otimes P) e^{-S} H' e^{S} (\mathrm{I} \otimes P) - H \otimes P\| \leq O(n\epsilon + n\eta J + n\eta^3 J'_O + n\eta^4 J') \ , \tag{122}$$

and by condition (118) this implies that

$$\frac{\tau}{\Delta} \to 0 \quad \text{as } n \to \infty. \tag{123}$$

Letting $\tilde{P} = e^{S} (\mathrm{I} \otimes P) e^{-S}$ and applying the triangle inequality, this gives

$$\|\tilde{P} H' \tilde{P}\| \leq \|H\| + \tau \ . \tag{124}$$

We seek to use Lemma 18 to argue that $\tilde{P}$ is "close to" $P_{\leq \Delta'(H')}$. To do this, we start by bounding $\|\tilde{P} H' \tilde{P}^{\perp}\|$. We have

$$\begin{aligned} \|\tilde{P} H' \tilde{P}^{\perp}\| &= \|(\mathrm{I} \otimes P) e^{-S} H' e^{S} (\mathrm{I} \otimes P^{\perp})\| \\ &\leq \sum_i \|(\mathrm{I} \otimes P) e^{-S} H'_i e^{S} (\mathrm{I} \otimes P^{\perp})\| \ , \end{aligned} \tag{125}$$

by the triangle inequality. For each i, we define $\tilde{H}_i = e^{-S_i} H'_i e^{S_i}$. Note that this operator is block diagonal in the basis of the projector $(\mathrm{I} \otimes P_i)$, in which S_i is block off-diagonal. Here we are using the definition of the direct rotation, and the fact that $e^{S_i} (\mathrm{I} \otimes P_i) e^{-S_i}$ is a low-energy projector for H'_i by definition. Note also that each $\tilde{H}_i$ acts on $O(1)$ sites. Now we can write

$$\|\tilde{P} H' \tilde{P}^{\perp}\| \leq \sum_i \|(\mathrm{I} \otimes P) e^{-S} e^{S_i} \tilde{H}_i e^{-S_i} e^{S} (\mathrm{I} \otimes P^{\perp})\| \ . \tag{126}$$

From here we use Lemma 11 to expand

$$e^{S_i} \tilde{H}_i e^{-S_i} = \tilde{H}_i + [S_i, \tilde{H}_i] + R_i \ , \tag{127}$$

where R_i acts on $O(1)$ sites, and by Lemma 12 we can bound $\|R_i\| = O(J' \eta^2)$. Hence

$$\begin{aligned} e^{-S} e^{S_i} \tilde{H}_i e^{-S_i} e^{S} = \tilde{H}_i &+ [S_i, \tilde{H}_i] + R_i \\ &- [S, \tilde{H}_i + [S_i, \tilde{H}_i] + R_i] \\ &+ \tilde{R}_i \ , \end{aligned} \tag{128}$$

where the remainder $\tilde{R}_i$ is similarly obtained by Lemmas 11-12 with $\|\tilde{R}_i\| = O(J'\eta^2)$. Then, using that S_i, $\tilde{H}_i$, and R_i each act on $O(1)$ sites, we can estimate

$$e^{-S}e^{S_i}\tilde{H}_i e^{-S_i}e^{S} = \tilde{H}_i - \sum_{j\neq i}[S_j, \tilde{H}_i] + O(J'\eta^2)\ . \tag{129}$$

Note that only $O(1)$ of the terms in the sum will be nonzero, and for $j \neq i$, S_j will commute with $\mathrm{I}\otimes P_i$. Therefore

$$\begin{aligned}\|(\mathrm{I}\otimes P)[S_j, \tilde{H}_i](\mathrm{I}\otimes P^{\perp})\| &\leq 2\|S_j\|\|(\mathrm{I}\otimes P_i)\tilde{H}_i\| \\ &\leq O(\eta)\|e^{-S_i}(\mathrm{I}\otimes P_i)e^{S_i}H'_i\| \\ &= O(\eta)\|P_{\leq\Delta(H'_i)}H'_i\| \\ &\leq O(\eta)(\|H_i\| + \epsilon) = O(\eta J + \eta\epsilon)\ . \end{aligned} \tag{130}$$

In the last line we have used the fact that H' defines a (Δ, η, ϵ)-gadget for H_i. Hence we can conclude that

$$(\mathrm{I}\otimes P)e^{-S}e^{S_i}\tilde{H}_i e^{-S_i}e^{S}(\mathrm{I}\otimes P^{\perp}) = O(J'\eta^2 + \eta J + \eta\epsilon)\ , \tag{131}$$

and so, inserting into (125), we have

$$\|\tilde{P}H'\tilde{P}^{\perp}\| := \omega = O(nJ'\eta^2 + n\eta J + n\eta\epsilon)\ . \tag{132}$$

Notice that, since we are interested in the case where $\epsilon = o(1)$ and $J = \theta(1)$, the first two terms on the right-hand side will typically dominate the third one.

Now we show that the restriction of H' to the image of $\tilde{P}^{\perp}$ has high-energy eigenvalues. Let $|\psi_{\mathcal{H}}\rangle\otimes|\psi_{\mathcal{A}_i}\rangle \in \mathcal{H}\otimes\mathcal{A}_i$. We consider the expression $(\langle\psi_{\mathcal{H}}|\otimes\langle\psi_{\mathcal{A}_i}|)H'_i(|\psi_{\mathcal{H}}\rangle\otimes|\psi_{\mathcal{A}_i}\rangle)$ in two cases:

- **Case 1:** $|\psi_{\mathcal{A}_i}\rangle \in P_i\mathcal{A}_i$

  Then

$$\begin{aligned}&(\langle\psi_{\mathcal{H}}|\otimes\langle\psi_{\mathcal{A}_i}|)H'_i(|\psi_{\mathcal{H}}\rangle\otimes|\psi_{\mathcal{A}_i}\rangle) \\ &\quad\geq (\langle\psi|_{\mathcal{H}}\otimes\langle\psi_{\mathcal{A}_i}|)P_{\leq\Delta(H'_i)}H'_iP_{\leq\Delta(H'_i)}(|\psi_{\mathcal{H}}\rangle\otimes|\psi_{\mathcal{A}_i}\rangle) \\ &\quad\geq (\langle\psi|_{\mathcal{H}}\otimes\langle\psi_{\mathcal{A}_i}|)e^{S_i}(H_i\otimes P_i)e^{-S_i}(|\psi_{\mathcal{H}}\rangle\otimes|\psi_{\mathcal{A}_i}\rangle) - \epsilon \\ &\quad\geq \langle\psi_{\mathcal{H}}|H_i|\psi_{\mathcal{H}}\rangle - (\epsilon + 2J\eta)\ . \end{aligned} \tag{133}$$

- **Case 2:** $|\psi_{\mathcal{A}_i}\rangle \in P_i^{\perp}\mathcal{A}_i$

  Then

$$\begin{aligned}&(\langle\psi_{\mathcal{H}}|\otimes\langle\psi_{\mathcal{A}_i}|)H'_i(|\psi_{\mathcal{H}}\rangle\otimes|\psi_{\mathcal{A}_i}\rangle) \\ &\quad\geq \Delta(\langle\psi_{\mathcal{H}}|\otimes\langle\psi_{\mathcal{A}_i}|)P_{>\Delta(H'_i)}(|\psi_{\mathcal{H}}\rangle\otimes|\psi_{\mathcal{A}_i}\rangle) \\ &\quad= \Delta\big((\langle\psi_{\mathcal{H}}|\otimes\langle\psi_{\mathcal{A}_i}|)e^{S_i}(\mathrm{I}\otimes P_i^{\perp})e^{-S_i}(|\psi_{\mathcal{H}}\rangle\otimes|\psi_{\mathcal{A}_i}\rangle)\big) \\ &\quad\geq \Delta(1-2\eta) \\ &\quad\geq \langle\psi_{\mathcal{H}}|H_i|\psi_{\mathcal{H}}\rangle\ , \end{aligned} \tag{134}$$

  using that $\Delta(1-2\eta) \geq J$ for large enough n, as $\Delta = \Omega(nJ)$ by assumption.

Now consider any $|\psi\rangle \in \mathcal{H} \otimes (\otimes_i \mathcal{A}_i)$ of the form

$$|\psi\rangle = e^S |\psi_{\mathcal{H}}\rangle \otimes (\otimes_i |\psi_{\mathcal{A}_i}\rangle) \ , \tag{135}$$

where $P_j|\psi_{\mathcal{A}_j}\rangle = 0$ for at least one value of j. Note that such states span the image of $\tilde{P}^\perp$. Then

$$\begin{aligned}
\langle\psi|\tilde{P}^\perp H' \tilde{P}^\perp|\psi\rangle &= \sum_i \langle\psi|H'_i|\psi\rangle \\
&\geq \sum_{i\neq j} \big(\langle\psi_{\mathcal{H}}|H_i|\psi_{\mathcal{H}}\rangle - \epsilon - 2J\eta\big) + \Delta(1-2\eta) \\
&\geq \langle\psi_{\mathcal{H}}|H|\psi_{\mathcal{H}}\rangle - (N-1)(\epsilon + 2J\eta) - J + \Delta(1-2\eta) \\
&\geq \Delta(1-2\eta) - \big(\|H\| + J + N(\epsilon + 2J\eta)\big) \\
&\geq \frac{3}{4}\Delta \ ,
\end{aligned} \tag{136}$$

where we have used the condition (116).

Now let $\tilde{H} := \tilde{P}H'\tilde{P} + \tilde{P}^\perp H' \tilde{P}^\perp$, so that $\|H' - \tilde{H}\| = O(nJ'\eta^2)$ by (132). Based on (124) and (136), we know that

$$\operatorname{spec} \tilde{H} \subset [-(\|H\| + \tau), \|H\| + \tau] \cup [\frac{3}{4}\Delta, \infty) \ , \tag{137}$$

corresponding to low- and high-energy projectors $\tilde{P}$ and $\tilde{P}^\perp$ respectively. Note that (116) in particular implies that $\Delta \geq 4\|H\|$, so $\frac{1}{2}\Delta - \|H\| \geq \frac{1}{4}\Delta$. Using this, and (123), we see

$$\frac{\frac{1}{2}\Delta - (\|H\| + \tau)}{\Delta} \geq \frac{1}{4} - \frac{\tau}{\Delta} = \Omega(1) \tag{138}$$

for large n. But by (116), (117), and (118),

$$\frac{\omega}{\Delta} = O(n\eta^2 J'/\Delta) + O(n\eta J/\Delta) + O(n\eta\epsilon/\Delta) = o(1) \ . \tag{139}$$

for sufficiently large n, we will have $\omega < \frac{1}{2}\Delta - (\|H\| + \tau)$, and hence

$$\operatorname{spec} \tilde{H} \subset (-\infty, \frac{1}{2}\Delta - \omega] \cup [\frac{3}{4}\Delta, \infty) \ , \tag{140}$$

once again corresponding to subspaces defined by $\tilde{P}$ and $\tilde{P}^\perp$. Now, by (132) and Lemma 4, we see that the full Hamiltonian H' has a ($\leq \frac{1}{2}\Delta$)-low energy subspace with the same dimension as the rank of $\tilde{P}$, and moreover

$$\operatorname{spec} H' \subset (-\infty, \frac{1}{2}\Delta] \cup [\frac{3}{4}\Delta - \omega, \infty) \ . \tag{141}$$

Now set $\Delta' = \Delta/2$. Notice that $\|\tilde{P} - P_{\leq\Delta'(H')}\| < 1$, since otherwise there would be a state of energy less than Δ' in the image of $\tilde{P}^\perp$, which is disallowed by (137). So the direct rotation $W \in \mathrm{U}(\mathcal{H} \otimes \mathcal{A})$ from $\tilde{P}$ to $P_{\leq\Delta'(H')}$ is well-defined, and by Lemma 18

$$\|W - \mathrm{I}\| \leq \frac{\sqrt{2}}{\frac{1}{2}\Delta - \|H\| - \tau}\omega \ . \tag{142}$$

Again using that $\Delta \geq 4\|H\|$, so $\frac{1}{2}\Delta - \|H\| \geq \frac{1}{4}\Delta = \Omega(\Delta)$. By (118), this will dominate the relatively

small τ term, so using (132) and that $\Delta = \Omega(J')$ we have

$$\begin{aligned}\|W - \mathrm{I}\,\| &= O(\omega/J') = O(n\eta^2 + n\eta J/J' + n\eta\epsilon/J') \\ &= O\big(n\eta^2(1 + J/\eta J' + \epsilon/J'\eta)\big) \\ &= O\Big(n\eta^2\big(1 + o(1/n) + o(\epsilon/nJ)\big)\Big) = O(n\eta^2)\ ,\end{aligned} \tag{143}$$

where in the last line we used (118). We can write W in terms of its anti-Hermitian and off-diagonal generator X, $W = e^X$, where $\|X\| = O(n\eta^2)$ by (38). Then

$$P_{\le\Delta'(H')} = U(\mathrm{I}\otimes P)U^\dagger\ , \tag{144}$$

where $U = e^X e^S$. Note that

$$\begin{aligned}\|U - \mathrm{I}\,\| &\le \|e^X(e^S - \mathrm{I})\| + \|e^X - \mathrm{I}\,\| \\ &\le \|S\| + \|X\| \\ &= O(n\eta) + O(n\eta^2) = O(n\eta)\ .\end{aligned} \tag{145}$$

It remains to bound find ϵ' to achieve a bound of the form

$$\|P_{\le\Delta'(H')}H'P_{\le\Delta'(H')} - U(H\otimes P)U^\dagger\| \le \epsilon'\ . \tag{146}$$

Using (124) and the triangle inequality we have

$$\|P_{\le\Delta'(H')}H'P_{\le\Delta'(H')} - U(H\otimes P)U^\dagger\| \le \|\tilde{P}(e^{-X}H'e^X - H')\tilde{P}\| + \tau\ , \tag{147}$$

and the first term can be bounded using Lemma 11 (and bounding the remainder with (56)) by

$$\begin{aligned}\|\tilde{P}(e^{-X}H'e^X - H')\tilde{P}\| &\le \|\tilde{P}[X, H']\tilde{P}\| + \frac{1}{2}\|[X,[X,H']]\| \\ &\le 2\|X\|\cdot\|\tilde{P}H'\tilde{P}^\perp\| + 2\|X\|^2\cdot\|H'\| \\ &\le O(n^3\eta^4 J' + n^2\eta^3 J)\ .\end{aligned} \tag{148}$$

In the second inequality we have used that X is off-diagonal with respect to $\tilde{P}$, and in the third inequality we have used (132) to bound $\|\tilde{P}H'\tilde{P}^\perp\|$. Hence

$$\epsilon' = O(n\epsilon + n\eta J + n\eta^3 J'_O + n^3\eta^4 J')\ . \tag{149}$$

■

4.3 Ground-state estimation with bounded-strength low-energy gadgets

Theorem 19 (Restatement of Theorem 25 in the Main Text)**.** *Let $H = \sum_i H_i$ be as in Setup 13,and suppose that each $(H_i', \mathcal{A}_i)$ defines a (Δ, η, ϵ)-gadget for H_i.*

Define

$$H' = \sum_i H'_i \in \mathrm{Herm}(\mathcal{H} \otimes (\otimes_i \mathcal{A}_i)) \ . \tag{150}$$

Then the ground state energies of H and H' satisfy

$$|\lambda_0(H) - \lambda_0(H')| = O(n\epsilon + n\eta J + n\eta^3 J'_O + n\eta^4 J') \ . \tag{151}$$

The following proof of Theorem 19 is a simple corollary of Proposition 14, and generalises the proof of Bravyi et al., Theorem 1[5].

Proof of Theorem 19. For the first part of this proof, we seek to put a lower bound on the individual gadgets H'_i. We write

$$H'_i = P_{\le\Delta(H'_i)} H'_i P_{\le\Delta(H'_i)} + P_{>\Delta(H'_i)} H'_i P_{>\Delta(H'_i)} \ , \tag{152}$$

and consider the high- and low-energy parts separately.

For the low-energy part, we can apply the gadget definition to write

$$\begin{aligned} \|P_{\le\Delta(H'_i)} H'_i P_{\le\Delta(H'_i)} - H_i \otimes P_i\| &\le \epsilon + \|e^{S_i}(H_i \otimes P_i)e^{-S_i} - H_i \otimes P_i\| \\ &\le O(\epsilon + \eta J) \ , \end{aligned} \tag{153}$$

using Lemma 11, and hence

$$P_{\le\Delta(H'_i)} H'_i P_{\le\Delta(H'_i)} \ge H_i \otimes P_i + O(\epsilon + \eta J) \ . \tag{154}$$

For the high-energy part, we first notice that since the spectrum of $P_{>\Delta(H'_i)} H'_i P_{>\Delta(H'_i)}$ lies in (Δ, ∞), where $\Delta \ge \|H_i\| - \epsilon$ (by the assumption of the (Δ, η, ϵ)-gadget definition that $P_{\le\Delta(H'_i)} H'_i P_{\le\Delta(H'_i)}$ has the same spectrum as H'_i up to error ϵ), and so

$$P_{>\Delta(H'_i)} H'_i P_{>\Delta(H'_i)} \ge P_{>\Delta(H'_i)} (H_i \otimes \mathrm{I}) P_{>\Delta(H'_i)} + O(\epsilon) \ . \tag{155}$$

Furthermore, we can approximate the RHS of this expression by

$$\begin{aligned} &\|P_{>\Delta(H'_i)} (H_i \otimes \mathrm{I}) P_{>\Delta(H'_i)} - H_i \otimes P_i^\perp\| \\ &\quad = \|e^{S_i}(\mathrm{I} \otimes P_i^\perp)e^{-S_i}(H_i \otimes \mathrm{I})e^{S_i}(\mathrm{I} \otimes P_i^\perp)e^{-S_i} - H_i \otimes P_i^\perp\| \\ &\quad \le O(\eta J) \ , \end{aligned} \tag{156}$$

by applying Lemma 11. Hence

$$P_{>\Delta(H'_i)} H'_i P_{>\Delta(H'_i)} \ge H_i \otimes P_i^\perp + O(\epsilon + \eta J) \ . \tag{157}$$

Summing (154) and (157) for all i, we obtain

$$H' = \sum_i H'_i \ge \sum_i (H_i \otimes \mathrm{I} + O(\epsilon + \eta J)) = H \otimes \mathrm{I} + O(n\epsilon + n\eta J) \ , \tag{158}$$

and so the ground state energy of H' must satisfy

$$\lambda_0(H') \ge \lambda_0(H) + O(n\epsilon + n\eta J) \ . \tag{159}$$

Now notice that the restriction of H' to a subspace can only increase λ_0, so

$$\lambda_0(H') = \lambda_0(e^{-S}H'e^{S}) \leq \lambda_0((\mathrm{I}\otimes P)e^{-S}H'e^{S}(\mathrm{I}\otimes P)) \ . \tag{160}$$

Here, as in the work of Bravyi et al.[5], we abuse notation slightly: in the expression $\lambda_0((\mathrm{I}\otimes P)e^{-S}H'e^{S}(\mathrm{I}\otimes P))$, we implicitly take the ground state of the restriction of $e^{-S}H'e^{S}$ to the image of $(\mathrm{I}\otimes P)$. So by Proposition 14 we have

$$\lambda_0(H') \leq \lambda_0(H) + O(n\epsilon + n\eta J + n\eta^3 J'_O + n\eta^4 J') \ . \tag{161}$$

Combining (159)-(161) gives the desired result. ■

Supplementary Note 5 — Gadget energy scaling

Theorem 20 (Restatement of Theorem 26 in the Main Text)**.** *Let $\mathcal{H} = (\mathbb{C}^2)^{\otimes k}$ be the space of $k = O(1)$ qubits, and let H be the k-fold tensor product of Pauli Z operators with strength $J > 0$,*

$$H = J\bigotimes_{i=1}^{k} Z_i \ . \tag{162}$$

Suppose $(H', \mathcal{A})$ is a (η, ϵ)-gadget for H for H' a k'-local Hamiltonian, where $k' < k$.

Then, provided $\epsilon < J$, the gadget must have energy scale $\|H'\| \geq \frac{J-\epsilon}{\eta} = \Omega(\eta^{-1})$.

In this section, we prove Theorem 20. Firstly, we introduce the notion of a k-local function, which can be thought of as a classical k-local observable on a state space $\{0,1\}^n$.

Definition 21 (k-local function)**.** Let $f : \{0,1\}^n \to \mathbb{R}$ be a function. We say that f is *k-local* if it can be written as a sum of functions

$$f(x_1, x_2, \ldots, x_n) = \sum_i f_i(x_1, x_2, \ldots, x_n) \ , \tag{163}$$

where the $f_i : \{0,1\}^n \to \mathbb{R}$ each depend on at most k of their inputs.

The following simple lemmas show that there exist k-local functions which cannot be approximated well by k'-local functions for $k' < k$.

Lemma 22. *Let f be a k-local function on n inputs. Then $\mathcal{R}f : \{0,1\}^{n-1} \to \mathbb{R}$, defined by*

$$\mathcal{R}f(x_1, \ldots, x_{n-1}) = f(x_1, \ldots, x_{n-1}, 0) - f(x_1, \ldots, x_{n-1}, 1) \tag{164}$$

is $(k-1)$-local.

Proof of Lemma 22. Decomposing $f = \sum_i f_i$ as in Definition 21, note that any f_i which does not depend on x_n has $\mathcal{R}f_i = 0$. Moreover, any f_i which does depend on x_n depends on at most $(k-1)$ other inputs, hence $\mathcal{R}f_i$ is $(k-1)$-local. ■

Lemma 23. *Let $k > k' > 0$. There exists a k-local function $f : \{0,1\}^k \to \mathbb{R}$ with $\max_{x\in\{0,1\}^k} |f(x)| \le 1$ such that for any k'-local function $g : \{0,1\}^k \to \mathbb{R}$,*

$$\max_{x\in\{0,1\}^k} |f(x) - g(x)| \ge 1 \ . \tag{165}$$

Proof of Lemma 23. For any $r \ge 1$, we can define $\mathcal{R}^r f : \{0,1\}^{k-r} \to \mathbb{R}$ by

$$\mathcal{R}^r f(x_1, \ldots, x_{k-r}) = \sum_{x_{k-r+1},\ldots,x_k \in\{0,1\}} (-1)^{\sum_{j=1}^r x_{k-r+j}} f(x_1, \ldots, x_k) \ . \tag{166}$$

Applying Lemma 22 inductively, note that $\mathcal{R}^r f$ is $(k-r)$-local. In particular, $\mathcal{R}^{k'} g$ is constant for any k'-local g.

Let $f : \{x_1, \ldots, x_k\}$ be the parity function

$$f(x_1, \ldots, x_k) = (-1)^{\sum_{i=1}^k x_i} \ . \tag{167}$$

Then we can calculate

$$\begin{aligned}\mathcal{R}^{k'} f(x_1, \ldots, x_{k-k'}) &= \sum_{x_{k-k'+1},\ldots,x_k \in\{0,1\}} (-1)^{\sum_{j=1}^{k'} x_{k-k'+j}} \cdot (-1)^{\sum_{i=1}^k x_i} \\ &= 2^{k'} (-1)^{\sum_{j=1}^{k-k'} x_j}\end{aligned} \tag{168}$$

Hence $\mathcal{R}^{k'} f(x_1, \ldots, x_{k-k'})$ takes values $\pm 2^{k'}$, whereas $\mathcal{R}^{k'} g$ is a constant function. So there must exist exist $y \in \{0,1\}^{k-k'}$ such that

$$|\mathcal{R}^{k'} f(y) - \mathcal{R}^{k'} g(y)| \ge 2^{k'} \ . \tag{169}$$

Hence, expanding $\mathcal{R}^{k'} f(y)$ and $\mathcal{R}^{k'} g(y)$ into $2^{k'}$ terms using (166), we must have

$$\max_{x\in\{0,1\}^k} |f(x) - g(x)| \ge 1 \ . \tag{170}$$

■

Note that Lemma 23 uses the parity function (which appears in the proof of Theorem 20) for illustration, but a similar argument could apply to most k-local functions; the vector space of k-local functions has a higher dimension than that of k'-local functions.

The following proof uses the intuition from Lemma 23 to argue that the target k-local term cannot be reproduced by a k'-local Hamiltonian.

Proof of Theorem 20. By the gadget definition, we have $U \in \mathrm{U}(\mathcal{H} \otimes \mathcal{A})$ and $P \in \mathrm{Proj}(\mathcal{A})$ such that

$$\|U - \mathrm{I}\| \le \eta \ , \quad \|P'H'P' - U(H \otimes P)U^\dagger\| \le \epsilon \ , \quad \text{where } P' = U(\mathrm{I} \otimes P)U^\dagger \ . \tag{171}$$

For any given $x \in \{0,1\}^k$, define $|\psi_x\rangle \in \mathcal{H}$ to be the pure state whose ith qubit is in the state $|x_i\rangle$.

Moreover let $|\phi\rangle \in \mathcal{A}$ be some state satisfying $P|\phi\rangle = |\phi\rangle$. Then define functions $F, f : \{0,1\}^k \to \mathbb{R}$ by

$$\begin{aligned} F(x) &= \mathrm{tr}[H|\psi_x\rangle\langle\psi_x|] \ , \\ f(x) &= \mathrm{tr}[H'\big(|\psi_x\rangle\langle\psi_x| \otimes |\phi\rangle\langle\phi|\big)] \ . \end{aligned} \tag{172}$$

Notice that F and f are k- and k'-local respectively, and by Lemma 23 there exists some $y \in \{0,1\}^k$ such that

$$|F(y) - f(y)| \geq J \ . \tag{173}$$

On the other hand, for all $x \in \{0,1\}^k$ we have

$$\begin{aligned} |F(x) - f(x)| &= |\,\mathrm{tr}[(H \otimes P)(|\psi_x\rangle\langle\psi_x| \otimes |\phi\rangle\langle\phi|)] - \mathrm{tr}[H'(|\psi_x\rangle\langle\psi_x| \otimes |\phi\rangle\langle\phi|)]| \\ &\leq \|(\mathrm{I} \otimes P)H'(\mathrm{I} \otimes P) - H \otimes P\| \\ &= \|P'UH'U^\dagger P' - U(H \otimes P)U^\dagger\| \\ &\leq \epsilon + \|P'(H' - UH'U^\dagger)P'\| \\ &\leq \epsilon + 2\eta\|H'\| \ . \end{aligned} \tag{174}$$

Hence we must have scaling

$$\|H'\| \geq \frac{J - \epsilon}{2\eta} \ , \tag{175}$$

as required. ∎

Supplementary Note 6 — Dissipative gadgets

6.1 Dissipative gadgets in isolation

Proposition 24 (Restatement of Proposition 27 in the Main Text)**.** *For a Hilbert space $\mathcal{H}$ and an ancillary qubit $\mathcal{A} = \mathbb{C}^2$, let $H' \in \mathrm{Herm}(\mathcal{H} \otimes \mathcal{A})$ be a Hamiltonian given by*

$$H' = H_{\mathrm{I}} \otimes \mathrm{I} + H_X \otimes X + H_{|1\rangle\langle 1|} \otimes |1\rangle\langle 1| \ , \tag{176}$$

for some $H_{\mathrm{I}}, H_X, H_{|1\rangle\langle 1|} \in \mathrm{Herm}(\mathcal{H})$ depending on a small parameter δt such that $\|H_{\mathrm{I}}\| = O(1)$, $\|H_X\| = O((\delta t)^{-1/2})$, and $\|H_{|1\rangle\langle 1|}\| = O((\delta t)^{-1})$ with $H_{|1\rangle\langle 1|}^2 = \omega^2\,\mathrm{I}$, $\omega = \frac{2\pi}{\delta t}$.

Then, for any $|\psi\rangle \in \mathcal{H}$,

$$e^{-i\delta t H'}(|\psi\rangle \otimes |0\rangle) = \big(e^{-i\delta t H}|\psi\rangle + O((\delta t)^2)\big) \otimes |0\rangle + O((\delta t)^{3/2}) \otimes |1\rangle \ , \tag{177}$$

where

$$H = H_{\mathrm{I}} - \omega^{-2} H_X H_{|1\rangle\langle 1|} H_X \ . \tag{178}$$

Here we first prove Proposition 24. This follows from direct calculation, by Taylor expanding the expression $e^{-i\delta t H'}$ and identifying the leading order terms in δt. This approach is complicated by the fact that H' itself consists of terms that are $O((\delta t)^{-1})$ and $O((\delta t)^{-1/2})$, but the task is simplified since we only need to calculate the time evolution of states of the form $|\psi\rangle \otimes |0\rangle$.

Proof of Proposition 24. First, notice that the requirement $H_{|1\rangle\langle 1|}^2 = \omega^2\,\mathrm{I}$ implies that

$$e^{-i\delta t H_{|1\rangle\langle 1|}} = \mathrm{I}\ , \quad H_{|1\rangle\langle 1|}^{-1} = \omega^{-2} H_{|1\rangle\langle 1|}\ . \tag{179}$$

Now we expand $e^{-i\delta t H'}$:

$$e^{-i\delta t H'} = \sum_{k\geq 0} \frac{(-i\delta t)^k}{k!} \big(H_{\mathrm{I}}\otimes \mathrm{I} + H_X \otimes X + H_{|1\rangle\langle 1|}\otimes |1\rangle\langle 1|\big)^k. \tag{180}$$

We can expand out this expression so that each term is a product of a factors of $H_{\mathrm{I}}\otimes\mathrm{I}$, b factors of $H_X\otimes X$, and c factors of $H_{|1\rangle\langle 1|}$, for some $a,b,c\in\mathbb{N}$. Such a term is accompanied by $(\delta t)^{a+b+c}$, to give a total order of $(\delta t)^{a+\frac{1}{2}b}$ (using that $\|H_X\| = O((\delta t)^{-1/2})$ and $\|H_{|1\rangle\langle 1|}\| = O((\delta t)^{-1})$). There are eight cases producing terms of order $O((\delta t)^{3/2})$ and lower, which we enumerate in Supplementary Figure 2.

Case	a	b	c	Order
1	0	0	0	$O(1)$
2	0	0	$\mathbb{N}_+$	
3	0	1	$\mathbb{N}$	$O((\delta t)^{1/2})$
4	1	0	0	$O(\delta t)$
5	1	0	$\mathbb{N}_+$	
6	0	2	$\mathbb{N}$	
7	1	1	$\mathbb{N}$	$O(t^{3/2})$
8	0	3	$\mathbb{N}$	

Supplementary Figure 2: Enumeration of possible cases for the values of a,b,c giving rise to terms of order $O((\delta t)^{3/2})$ and lower in (180). We denote $\mathbb{N}_+ = \{1,2,\dots\}$ and $\mathbb{N} = \{0,1,2,\dots\}$.

In particular, we are interested in the block-elements $(\mathrm{I}\otimes\langle 0|)e^{-i\delta t H'}(\mathrm{I}\otimes|0\rangle)$ and $(\mathrm{I}\otimes\langle 1|)e^{-i\delta t H'}(\mathrm{I}\otimes|0\rangle)$, since the other blocks in $e^{-i\delta t H'}$ will annihilate states of the form $|\psi\rangle\otimes|0\rangle$.

- $(\mathrm{I}\otimes\langle 0|)e^{-i\delta t H'}(\mathrm{I}\otimes|0\rangle)$:

  Note that each factor of $H_X\otimes X$ flips the ancillary qubit $\mathcal{A}$, whereas each factor of $H_{|1\rangle\langle 1|}\otimes|1\rangle\langle 1|$ annihilates states with the ancillary qubit in state $|0\rangle$. As a result, the only contributions to $(\mathrm{I}\otimes\langle 0|)e^{-i\delta t H'}(\mathrm{I}\otimes|0\rangle)$ from Supplementary Figure 2 are those such that b is even. Moreover, c can only be nonzero if b is at least 2 (so that the factors of $H_{|1\rangle\langle 1|}\otimes|1\rangle\langle 1|$ can be sandwiched between two $H_X\otimes X$ factors). This restricts us to cases 1, 4, and 6, so

$$(\mathrm{I}\otimes\langle 0|)e^{-i\delta t H'}(\mathrm{I}\otimes|0\rangle) = \mathrm{I} - i\delta t H_{\mathrm{I}} + \sum_{k\geq 2}\frac{(-i\delta t)^k}{k!} H_X H_{|1\rangle\langle 1|}^{k-2} H_X + O((\delta t)^2)\ . \tag{181}$$

  Furthermore, the sum can be simplified to

$$\begin{aligned}\sum_{k\geq 2}\frac{(-i\delta t)^k}{k!} H_X H_{|1\rangle\langle 1|}^{k-2} H_X &= H_X H_{|1\rangle\langle 1|}^{-2}\Big(\sum_{k\geq 2}\frac{(-i\delta t)^k}{k!} H_{|1\rangle\langle 1|}^{k}\Big) H_X \\ &= H_X H_{|1\rangle\langle 1|}^{-2}\Big(e^{-i\delta t H_{|1\rangle\langle 1|}} - \mathrm{I} + i\delta t H_{|1\rangle\langle 1|}\Big) H_X \\ &= i\delta t\,\omega^{-2} H_X H_{|1\rangle\langle 1|} H_X\ ,\end{aligned} \tag{182}$$

using (179). Hence we have shown that

$$(\mathrm{I}\otimes\langle 0|)e^{-i\delta t H'}(\mathrm{I}\otimes|0\rangle) = \mathrm{I} - i\delta t(H_{\mathrm{I}} - \omega^{-2}H_X H_{|1\rangle\langle 1|}H_X) + O((\delta t)^2)\ . \tag{183}$$

- $(\mathrm{I}\otimes\langle 1|)e^{-i\delta t H'}(\mathrm{I}\otimes|0\rangle)$:

  By a similar argument to above, the only contributing terms from Supplementary Figure 2 are those such that b is odd, so we can reduce to the cases 3, 7, and 8. In case 3, also notice that the $H_X\otimes X$ term must appear on the right of all the $H_{|1\rangle\langle 1|}\otimes|1\rangle\langle 1|$ terms. Hence

$$(\mathrm{I}\otimes\langle 1|)e^{-i\delta t H'}(\mathrm{I}\otimes|0\rangle) = \sum_{k\geq 1}\frac{(-i\delta t)^k}{k!}H_{|1\rangle\langle 1|}^{k-1}H_X + O((\delta t)^{3/2})\ . \tag{184}$$

  The sum here can be similarly simplified by (179):

$$\begin{aligned}\sum_{k\geq 1}\frac{(-i\delta t)^k}{k!}H_{|1\rangle\langle 1|}^{k-1}H_X &= H_{|1\rangle\langle 1|}^{-1}\left(\sum_{k\geq 0}\frac{(-i\delta t)^k}{k!}H_{|1\rangle\langle 1|}^{k} - \mathrm{I}\right)H_X\\ &= H_{|1\rangle\langle 1|}^{-1}\left(e^{-i\delta t H_{|1\rangle\langle 1|}} - \mathrm{I}\right)H_X\\ &= 0\ ,\end{aligned} \tag{185}$$

  so

$$(\mathrm{I}\otimes\langle 1|)e^{-i\delta t H'}(\mathrm{I}\otimes|0\rangle) = O((\delta t)^{3/2})\ , \tag{186}$$

  which, along with (183), completes the proof.

■

6.2 Trotter errors

Proposition 25 (Restatement of Proposition 28 in the Main Text)**.** *Let $H_{else} = \sum_i h_i$ be a k-local Hamiltonian on $\mathcal{H} = \otimes_i\mathcal{H}_i$ such that $\|h_i\| = O(1)$, and whose interaction graph has a degree bounded by an $O(1)$ constant.*

Introduce an ancillary qubit $\mathcal{A} = \mathbb{C}^2$, and let $H' \in \mathrm{Herm}(\mathcal{H}\otimes\mathcal{A})$ be a Hamiltonian given by

$$H' = H_{\mathrm{I}}\otimes\mathrm{I} + H_X\otimes X + H_{|1\rangle\langle 1|}\otimes|1\rangle\langle 1|\ , \tag{187}$$

for some $H_{\mathrm{I}}, H_X, H_{|1\rangle\langle 1|} \in \mathrm{Herm}(\mathcal{H})$ depending on a small parameter δt such that $\|H_{\mathrm{I}}\| = O(1)$, $\|H_X\| = O((\delta t)^{-1/2})$, and $\|H_{|1\rangle\langle 1|}\| = O((\delta t)^{-1})$ with $H_{|1\rangle\langle 1|}^2 = \omega^2\,\mathrm{I}$, $\omega = \frac{2\pi}{\delta t}$. Assume that H_{I}, H_X, and $H_{|1\rangle\langle 1|}$ act on $O(1)$ sites in $\mathcal{H}$.

Then, for any $|\psi\rangle\in\mathcal{H}$,

$$e^{-i\delta t(H' + H_{else}\otimes\mathrm{I})}(|\psi\rangle\otimes|0\rangle) = \left(e^{-i\delta t(H+H_{else})}|\psi\rangle + O((\delta t)^2)\right)\otimes|0\rangle + O((\delta t)^{3/2})\otimes|1\rangle\ , \tag{188}$$

where

$$H = H_{\mathrm{I}} - \omega^{-2}H_X H_{|1\rangle\langle 1|}H_X\ . \tag{189}$$

(Proof on page 29.)

The idea for the proof of Proposition 25 is to factorise the overall evolution operator

$$e^{-i\delta t(H'+H_{\text{else}}\otimes \mathrm{I})} \approx e^{-i\delta t H_{\text{else}}\otimes \mathrm{I}} e^{-i\delta t H'} \ . \tag{190}$$

From here, we simply apply Proposition 24 to the initial state $|\psi\rangle \otimes |0\rangle$, and then evolve the $\mathcal{H}$ system of the resultant state under H_{else}. The technical difficulty is in bounding the errors of this Trotter expansion in a way which does not depend on the size of the system. Qualitatively, one might expect this behaviour due to the bounded spread of correlations in the system over a short time δt, under which only a limited set of interactions in H_{else} can "interfere" with the evolution under H'. The difficulty of obtaining such bounds is compounded by the presence of terms in H' which scale as $O((\delta t)^{-1})$ and $O((\delta t)^{-1/2})$. Our approach uses an explicit form of the Trotter error given by Childs et al.[7]. We briefly outline this process here.

Let $A, B \in \mathrm{Lin}(\mathcal{H})$. We aim to find an expression for the Trotter error incurred by the expansion $e^{t(A+B)} \approx e^{tA}e^{tB}$.

Observe that the function $f(t) = e^{tA}e^{tB}$ satisfies the differential equation

$$\begin{aligned} f'(t) &= Ae^{tA}e^{tB} + e^{tA}Be^{tB} \\ &= (A+B)f(t) + e^{tA}\big(B - e^{-tA}Be^{tA}\big)e^{tB} \ . \end{aligned} \tag{191}$$

This differential equation, with initial condition $f(0) = \mathrm{I}$, can be solved using following lemma.

Lemma 26 (Variation of parameters formula[7])**.** *Let $K \in \mathrm{Lin}(\mathcal{H})$, and let $L(t) \in \mathrm{Lin}(\mathcal{H})$ be a continuous operator-valued function of t. Suppose that $f(t)$ satisfies the differential equation*

$$f'(t) = Kf(t) + L(t) \ , \quad f(0) = \mathrm{I} \ . \tag{192}$$

Then there is a unique solution for f which is given by

$$f(t) = e^{tK} + \int_0^t \mathrm{d}\tau e^{(t-\tau)K} L(\tau) \ . \tag{193}$$

Hence, using Lemma 26 with $K = A + B$ and $L(t) = e^{tA}(B - e^{-tA}Be^{tA})e^{tB}$, we find that the Trotter error is given by

$$e^{tA}e^{tB} - e^{t(A+B)} = \int_0^t \mathrm{d}\tau e^{(t-\tau)(A+B)} e^{\tau A}\big(B - e^{-\tau A}Be^{\tau A}\big)e^{\tau B} \tag{194}$$

The expression (194) is particularly convenient because, when A is a local Hamiltonian and B acts only on $O(1)$ sites, the bracketed term $(B - e^{-\tau A}Be^{\tau A})$ can be bounded independently of n.

Lemma 27. *Let $H = \sum_i h_i$ be a k-local Hamiltonian on a system $\mathcal{H} = \otimes_{i=1}^n \mathcal{H}_i$, with the degree of the interaction hypergraph bounded by an $O(1)$ constant and $\|h_i\| = O(1)$. Let A be an observable supported on a set of $O(1)$ sites. Then*

$$\|e^{itH}Ae^{-itH} - A\| \leq O(\|A\|t) \ . \tag{195}$$

Proof of Lemma 27. For $X \in \mathrm{Lin}(\mathcal{H})$, define $f_X(t) = \mathrm{tr}[X(e^{itH}Ae^{-itH} - A)]$, so that

$$\|e^{itH}Ae^{-itH} - A\| = \max_{X \in D(\mathcal{H})} |f_X(t)| \ . \tag{196}$$

We can see that $f_X(0) = 0$ and $f'_X(t) = i\,\mathrm{tr}[Xe^{itH}[H,A]e^{-itH}]$. Moreover, since H is local on an $O(1)$-degree hypergraph, and A is supported on an $O(1)$ set, only $O(1)$ terms in H contribute to the commutator and hence $|f'_X(t)| \leq \|X\|_1 \|[H,A]\| = O(\|X\|_1\|A\|)$. By the mean value theorem, we therefore deduce that

$$f_X(t) = O(\|X\|_1\|A\|t) \;, \tag{197}$$

so by (196) we are done. ∎

6.3 Dissipative gadgets with other terms

Proof of Proposition 25. Using (194) with $t = \delta t$, $A = -iH_{\text{else}} \otimes \mathrm{I}$, and $B = -iH'$, we obtain a Trotter error given by

$$\begin{aligned} &e^{-i\delta t H_{\text{else}}\otimes\mathrm{I}} e^{-i\delta t H'} - e^{-i\delta t(H_{\text{else}}\otimes\mathrm{I} + H')} := E \\ &\quad = -i\int_0^{\delta t} \mathrm{d}\,\tau e^{-i(\delta t-\tau)(H_{\text{else}}\otimes\mathrm{I}+H')} e^{-i\tau H_{\text{else}}\otimes\mathrm{I}} \big(H' - e^{i\tau H_{\text{else}}\otimes\mathrm{I}} H' e^{-i\tau H_{\text{else}}\otimes\mathrm{I}}\big) e^{-i\tau H'} \;. \end{aligned} \tag{198}$$

We can write E in block form in the basis of the ancillary space,

$$E = \begin{pmatrix} (\mathrm{I}\otimes\langle 0|)E(\mathrm{I}\otimes|0\rangle) & (\mathrm{I}\otimes\langle 0|)E(\mathrm{I}\otimes|1\rangle) \\ (\mathrm{I}\otimes\langle 1|)E(\mathrm{I}\otimes|0\rangle) & (\mathrm{I}\otimes\langle 1|)E(\mathrm{I}\otimes|1\rangle) \end{pmatrix} \;, \tag{199}$$

and we will focus on individually bounding these blocks. Notice that, commuting projectors on the ancillary space past $H_{\text{else}} \otimes \mathrm{I}$ and applying Lemma 27, we have

$$\begin{aligned} &\|(\mathrm{I}\otimes\langle 0|)(H' - e^{i\tau H_{\text{else}}\otimes\mathrm{I}} H' e^{-i\tau H_{\text{else}}\otimes\mathrm{I}})(\mathrm{I}\otimes|0\rangle)\| \\ &\quad = \|(\mathrm{I}\otimes\langle 0|)H'(\mathrm{I}\otimes|0\rangle) - e^{i\tau H_{\text{else}}}(\mathrm{I}\otimes\langle 0|)H'(\mathrm{I}\otimes|0\rangle)e^{-i\tau H_{\text{else}}}\| \\ &\quad = \|H_{\mathrm{I}} - e^{i\tau H_{\text{else}}} H_{\mathrm{I}} e^{-i\tau H_{\text{else}}}\| = O(\delta t) \;. \end{aligned} \tag{200}$$

With a similar process for the other blocks, we obtain the following bounds:

$$\|(\mathrm{I}\otimes\langle 0|)(H' - e^{i\tau H_{\text{else}}\otimes\mathrm{I}} H' e^{-i\tau H_{\text{else}}\otimes\mathrm{I}})(\mathrm{I}\otimes|0\rangle)\| = O(\delta t) \;, \tag{201a}$$

$$\|(\mathrm{I}\otimes\langle 1|)(H' - e^{i\tau H_{\text{else}}\otimes\mathrm{I}} H' e^{-i\tau H_{\text{else}}\otimes\mathrm{I}})(\mathrm{I}\otimes|0\rangle)\| = O(\delta t^{1/2}) \;, \tag{201b}$$

$$\|(\mathrm{I}\otimes\langle 0|)(H' - e^{i\tau H_{\text{else}}\otimes\mathrm{I}} H' e^{-i\tau H_{\text{else}}\otimes\mathrm{I}})(\mathrm{I}\otimes|1\rangle)\| = O(\delta t^{1/2}) \;, \tag{201c}$$

$$\|(\mathrm{I}\otimes\langle 1|)(H' - e^{i\tau H_{\text{else}}\otimes\mathrm{I}} H' e^{-i\tau H_{\text{else}}\otimes\mathrm{I}})(\mathrm{I}\otimes|1\rangle)\| = O(1) \;. \tag{201d}$$

Since our initial state is of the form $|\psi\rangle \otimes |0\rangle$, we need only bound the magnitudes of the blocks $(\mathrm{I}\otimes\langle 0|)E(\mathrm{I}\otimes|0\rangle)$ and $(\mathrm{I}\otimes\langle 1|)E(\mathrm{I}\otimes|0\rangle)$. To this end, we need to describe the action of the operator $e^{-i\tau H'}$ on the operators $(\mathrm{I}\otimes|0\rangle)$ and $(\mathrm{I}\otimes|1\rangle)$, for $0 \leq \tau \leq \delta t$. By considering the series expansion as in (180), and noting that the ancillary qubit can only be flipped by a $H_X \otimes X$ term of order $O((\delta t)^{1/2})$, we see that

$$e^{-i\tau H'}(\mathrm{I}\otimes|0\rangle) = O(1)\otimes|0\rangle + O((\delta t)^{1/2})\otimes|1\rangle \;, \tag{202a}$$

$$e^{-i\tau H'}(\mathrm{I}\otimes|1\rangle) = O((\delta t)^{1/2})\otimes|0\rangle + O(1)\otimes|1\rangle \;. \tag{202b}$$

Notice that here we abuse big-O notation for matrices; for example, in the above expression $O((\delta t)^{1/2})$ should be interpreted as a matrix with operator norm bounded by $O(\delta t^{1/2})$. We can also crudely upper

bound E as follows:

$$\begin{aligned}\|E\| &\leq \int_0^{\delta t} \mathrm{d}\,\tau \|H' - e^{i\tau H_{\mathrm{else}}\otimes \mathrm{I}} H' e^{-i\tau H_{\mathrm{else}}\otimes \mathrm{I}}\| \\ &\leq \int_0^{\delta t} \mathrm{d}\,\tau O(1) = O(\delta t) \ ,\end{aligned} \tag{203}$$

using (201d) to give the most pessimistic bound. Therefore in particular

$$e^{i\tau H_{\mathrm{else}}\otimes \mathrm{I}} e^{i(\delta t-\tau)(H_{\mathrm{else}}\otimes \mathrm{I}+H')} = e^{i\delta t H_{\mathrm{else}}\otimes \mathrm{I}} e^{i(\delta t-\tau)H'} + O(\delta t) \ , \tag{204}$$

so, using (202a),

$$\begin{aligned}e^{i\tau H_{\mathrm{else}}\otimes \mathrm{I}} e^{i(\delta t-\tau)(H_{\mathrm{else}}\otimes \mathrm{I}+H')}(\mathrm{I}\otimes|0\rangle) &= e^{-i\delta t H_{\mathrm{else}}\otimes \mathrm{I}} e^{i(\delta t-\tau)H'}(\mathrm{I}\otimes|0\rangle) + O(\delta t) \\ &= O(1)\otimes|0\rangle + O((\delta t)^{1/2})\otimes|1\rangle \ ,\end{aligned} \tag{205}$$

and similarly

$$e^{i\tau H_{\mathrm{else}}\otimes \mathrm{I}} e^{i(\delta t-\tau)(H_{\mathrm{else}}\otimes \mathrm{I}+H')}(\mathrm{I}\otimes|1\rangle) = O((\delta t)^{1/2})\otimes|0\rangle + O(1)\otimes|1\rangle \ . \tag{206}$$

We can now obtain the necessary bounds on the blocks of E. Firstly, we have

$$\begin{aligned}&\|(\mathrm{I}\otimes\langle 0|)E(\mathrm{I}\otimes|0\rangle)\| \\ &\quad\leq \int_0^{\delta t} \mathrm{d}\,\tau \|(\mathrm{I}\otimes\langle 0|)e^{-i(\delta t-\tau)(H_{\mathrm{else}}\otimes \mathrm{I}+H')} e^{-i\tau H_{\mathrm{else}}\otimes \mathrm{I}}(H' - e^{i\tau H_{\mathrm{else}}\otimes \mathrm{I}} H' e^{-i\tau H_{\mathrm{else}}\otimes \mathrm{I}}) e^{-i\tau H'}(\mathrm{I}\otimes|0\rangle)\| \\ &\quad\leq \int_0^{\delta t} \mathrm{d}\,\tau \Big[O(1)\|(\mathrm{I}\otimes\langle 0|)(H' - e^{i\tau H_{\mathrm{else}}\otimes \mathrm{I}} H' e^{-i\tau H_{\mathrm{else}}\otimes \mathrm{I}})(\mathrm{I}\otimes|0\rangle)\| \\ &\qquad + O((\delta t)^{1/2})\|(\mathrm{I}\otimes\langle 1|)(H' - e^{i\tau H_{\mathrm{else}}\otimes \mathrm{I}} H' e^{-i\tau H_{\mathrm{else}}\otimes \mathrm{I}})(\mathrm{I}\otimes|0\rangle)\| \\ &\qquad + O((\delta t)^{1/2})\|(\mathrm{I}\otimes\langle 0|)(H' - e^{i\tau H_{\mathrm{else}}\otimes \mathrm{I}} H' e^{-i\tau H_{\mathrm{else}}\otimes \mathrm{I}})(\mathrm{I}\otimes|1\rangle)\| \\ &\qquad + O(\delta t)\|(\mathrm{I}\otimes\langle 1|)(H' - e^{i\tau H_{\mathrm{else}}\otimes \mathrm{I}} H' e^{-i\tau H_{\mathrm{else}}\otimes \mathrm{I}})(\mathrm{I}\otimes|1\rangle)\|\Big] \\ &\quad= O((\delta t)^2) \ ,\end{aligned} \tag{207}$$

using (201a-201d). Similarly, we can bound

$$\begin{aligned}&\|(\mathrm{I}\otimes\langle 1|)E(\mathrm{I}\otimes|0\rangle)\| \\ &\quad\leq \int_0^{\delta t} \mathrm{d}\,\tau \|(\mathrm{I}\otimes\langle 1|)e^{-i(\delta t-\tau)(H_{\mathrm{else}}\otimes \mathrm{I}+H')} e^{-i\tau H_{\mathrm{else}}\otimes \mathrm{I}}(H' - e^{i\tau H_{\mathrm{else}}\otimes \mathrm{I}} H' e^{-i\tau H_{\mathrm{else}}\otimes \mathrm{I}}) e^{-i\tau H'}(\mathrm{I}\otimes|0\rangle)\| \\ &\quad\leq \int_0^{\delta t} \mathrm{d}\,\tau \Big[O((\delta t)^{1/2})\|(\mathrm{I}\otimes\langle 0|)(H' - e^{i\tau H_{\mathrm{else}}\otimes \mathrm{I}} H' e^{-i\tau H_{\mathrm{else}}\otimes \mathrm{I}})(\mathrm{I}\otimes|0\rangle)\| \\ &\qquad + O(1)\|(\mathrm{I}\otimes\langle 1|)(H' - e^{i\tau H_{\mathrm{else}}\otimes \mathrm{I}} H' e^{-i\tau H_{\mathrm{else}}\otimes \mathrm{I}})(\mathrm{I}\otimes|0\rangle)\| \\ &\qquad + O(\delta t)\|(\mathrm{I}\otimes\langle 0|)(H' - e^{i\tau H_{\mathrm{else}}\otimes \mathrm{I}} H' e^{-i\tau H_{\mathrm{else}}\otimes \mathrm{I}})(\mathrm{I}\otimes|1\rangle)\| \\ &\qquad + O((\delta t)^{1/2})\|(\mathrm{I}\otimes\langle 1|)(H' - e^{i\tau H_{\mathrm{else}}\otimes \mathrm{I}} H' e^{-i\tau H_{\mathrm{else}}\otimes \mathrm{I}})(\mathrm{I}\otimes|1\rangle)\|\Big] \\ &\quad= O((\delta t)^{3/2}) \ .\end{aligned} \tag{208}$$

With the bounds (207) and (208) on the blocks of the Trotter error we can now conclude that

$$\begin{aligned} e^{-i\delta t(H'+H_{\text{else}}\otimes \mathrm{I})}(|\psi\rangle \otimes |0\rangle) &= e^{-i\delta t H_{\text{else}}\otimes \mathrm{I}} e^{-i\delta t H'}(|\psi\rangle \otimes |0\rangle) + E(|\psi\rangle \otimes |0\rangle) \\ &= e^{-i\delta t H_{\text{else}}\otimes \mathrm{I}}(e^{-i\delta t H}|\psi\rangle + O((\delta t)^2)) \otimes |0\rangle + O((\delta t)^{3/2}) \otimes |1\rangle \\ &\quad + O((\delta t)^2) \otimes |0\rangle + O((\delta t)^{3/2}) \otimes |1\rangle \\ &= \left(e^{-i\delta t H_{\text{else}}} e^{-i\delta t H}|\psi\rangle + O((\delta t)^2)\right) \otimes |0\rangle + O((\delta t)^{3/2}) \otimes |1\rangle \ , \end{aligned} \tag{209}$$

where in the second inequality we invoke Proposition 24. It remains only to bound the Trotter error in the product $e^{-i\delta t H_{\text{else}}} e^{-i\delta t H}$, which we can accomplish similarly. Using (194) with $t = \delta t$, $A = -iH_{\text{else}}$, $B = -iH$, we obtain

$$\begin{aligned} &e^{-i\delta t H_{\text{else}}} e^{-i\delta t H} - e^{-i\delta t(H+H_{\text{else}})} \\ &\quad = -i \int_0^{\delta t} \mathrm{d}\,\tau e^{-i(\delta t-\tau)(H+H_{\text{else}})} e^{-i\tau H_{\text{else}}}(H - e^{i\tau H_{\text{else}}} H e^{-i\tau H_{\text{else}}}) e^{-i\tau H} \ . \end{aligned} \tag{210}$$

So by Lemma 27 we have

$$e^{-i\delta t H_{\text{else}}} e^{-i\delta t H} - e^{-i\delta t(H+H_{\text{else}})} \leq O((\delta t)^2) \tag{211}$$

Combining (211) with (209) completes the proof. ■

References

[1] Toby S. Cubitt, Ashley Montanaro, and Stephen Piddock. Universal quantum Hamiltonians. *Proceedings of the National Academy of Sciences*, 115(38):9497–9502, 2018.

[2] Rajendra Bhatia. *Matrix analysis*, volume 169. Springer Science and Business Media, 2013.

[3] Sergey Bravyi, David P. DiVincenzo, and Daniel Loss. Schrieffer–Wolff transformation for quantum many-body systems. *Annals of Physics*, 326(10):2793–2826, 2011.

[4] Chandler Davis and William M. Kahan. Some new bounds on perturbation of subspaces. *Bulletin of the American Mathematical Society*, 75(4):863–868, 1969.

[5] Sergey Bravyi, David P. DiVincenzo, Daniel Loss, and Barbara M. Terhal. Quantum simulation of many-body Hamiltonians using perturbation theory with bounded-strength interactions. *Physical Review Letters*, 101(7):070503, 2008.

[6] Chandler Davis and William Morton Kahan. The rotation of eigenvectors by a perturbation. III. *SIAM Journal on Numerical Analysis*, 7(1):1–46, 1970.

[7] Andrew M. Childs, Yuan Su, Minh C. Tran, Nathan Wiebe, and Shuchen Zhu. Theory of Trotter error with commutator scaling. *Physical Review X*, 11(1):011020, 2021.
